# Exploring the effect of activator topology on CRISPR–Cas12a *trans*-cleavage activity

**DOI:** 10.1093/nar/gkaf311

**Published:** 2025-04-22

**Authors:** Zixuan Zhu, Xiaolong Li, Lin Ding, Tongbo Wu

**Affiliations:** School of Pharmacy, Tongji Medical College, Huazhong University of Science and Technology, Wuhan 430030, China; School of Pharmacy, Tongji Medical College, Huazhong University of Science and Technology, Wuhan 430030, China; School of Pharmacy, Tongji Medical College, Huazhong University of Science and Technology, Wuhan 430030, China; School of Pharmacy, Tongji Medical College, Huazhong University of Science and Technology, Wuhan 430030, China

## Abstract

The CRISPR–Cas12a system is widely used in nucleic acid detection and biosensing due to its high sensitivity, selectivity, and simple design. However, traditional CRISPR–Cas12a sensors, which rely on linear activators, face challenges such as limited operability and low stability. This study explored the impact of three different activator topologies—linear, planar, and steric—on the *trans*-cleavage activity of Cas12a. We developed a Cas12a-based switch using a planar activator, which demonstrated superior operability and maintained higher activity compared to linear activators. Using this planar activator, we achieved highly sensitive detection of hypochlorous acid, with a detection limit as low as 88 nM, outperforming chemical probe-based methods. The introduction of topological activators will open new avenues for the development of CRISPR–Cas12a-based biosensors, offering broad potential for diverse applications.

## Graphical Abstract

**Graphical Abstract:**
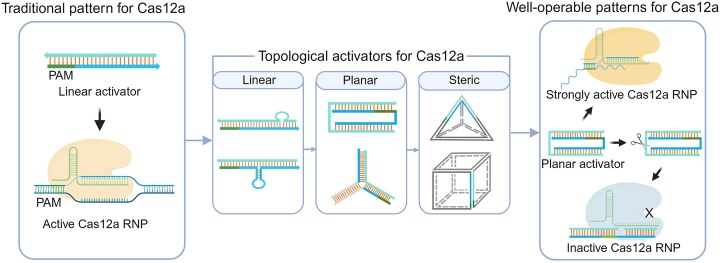

## Introduction

The clustered regularly interspaced short palindromic repeats (CRISPR) system is an adaptive immune mechanism found in prokaryotic cells [1]. It consists of CRISPR arrays and CRISPR-associated (Cas) proteins, which work together to recognize and cleave viral nucleic acids that invade bacteria [2]. Cas12a, a Class II CRISPR effector protein, is one of the most widely used proteins in the CRISPR–Cas system [3]. It targets both single-stranded DNA (ssDNA) and double-stranded DNA (dsDNA) under the guidance of CRISPR RNA (crRNA), facilitating gene cleavage and the generation of sticky ends [4, 5]. After cleavage, the RuvC domain at the cleavage site remains open, allowing further hydrolysis of any ssDNA, a property known as *trans*-cleavage activity [6, 7]. This versatility makes Cas12a highly applicable in various fields, including nucleic acid detection [8–10], mutation analysis [11, 12], biosensing [13, 14], and cell imaging [15, 16]. Most studies involving Cas12a have focused on linear DNA activators, with limited investigations into the effects of activator topology. Linear activators, however, present challenges, such as low maneuverability and instability in physiological environments, which hinder the development of robust biosensors and limit the broader application of Cas12a.

Conventional activation of Cas12a typically involves ssDNA or dsDNA, although some studies have explored alternative activators, such as RNA [17, 18] or split DNA [19, 20]. Notably, research on the activation of Cas12a by activators with different topologies remains limited. In 2015, Zhang *et al.* [3] characterized the gene-editing abilities of different Cas12a subtypes and verified their activity *in vitro*. In 2022, Nie *et al.* [21] demonstrated that a bubble-structured dsDNA activator could activate Cas12a without requiring the protospacer adjacent motif (PAM) sequence. In 2023, Kong *et al.* [22] introduced a special activation mode called “RESET”, in which an activator with a stem–loop structure at the 5′ end of a ssDNA enhances Cas12a activation. In 2024, Glodys *et al.* [23] reported that cyclic DNA structures could act as topological barriers to Cas12a; short-stranded cyclic dsDNA or ssDNA exhibited weaker affinity for Cas12a and inhibited its activity. Despite these advancements, the activator topology preferences of Cas12a remain to be thoroughly explored to expand its potential applications.

In this study, we designed linear, planar, and steric activator topologies. We systematically investigated how these different activator topologies affect the *trans*-cleavage activity of Cas12a at multiple levels. Building on these findings, we designed phosphorothioate-modified planar activators and developed two hypochlorous acid (HOCl) assays based on allosteric regulation. Our results provide promising activator designs that simplify the development of Cas12a-based biosensors and demonstrate broad potential for future applications.

## Materials and methods

### Chemicals and materials

All oligonucleotides used in this study (sequences in Supplementary Table S1) were synthesized and purified by Sangon Biotech Co., Ltd (Shanghai, China). EnGen Lba Cas12a (Cpf1, LbCas12a), 10× NEBuffer r3.1 (1 M NaCl, 500 mM Tris–HCl, 100 mM MgCl_2_, 1 mg/ml recombinant albumin, pH 7.9 at 25°C), 10× NEBuffer r2.1 (100 mM Tris–HCl, 100 mM MgCl_2_, 500 mM NaCl, 1 mg/ml recombinant albumin, pH 7.9 at 25°C), and 10× NEBuffer 4 (200 mM Tris–acetate, 100 mM magnesium acetate, 500 mM potassium acetate, 10 mM dithiothreitol, pH 7.9 at 25°C) were brought from New England Biolabs Inc. (Beijing, China). Deionized water (DNase/RNase-free) was obtained from Tiangen Biotech (Beijing, China) and used in all experiments. Sulfuric acid and sodium hypochlorite aqueous solution were purchased from Sinopharm Chemical Reagent Co., Ltd (Shanghai, China). 5× Tris-borate EDTA(TBE) buffer, 4S Red Plus Nucleic Acid Stain, 6× DNA Loading Dye, and DNAMarker A (25–500 bp), Native PAGE Preparation Kit were purchased from Sangon Biotech Co., Ltd (Shanghai, China). The concentrations of DNA/RNA oligonucleotides were measured by NanoDrop 2000 UV–Vis Spectrophotometer (Thermo Fisher Scientific, MA, USA). The fluorescence (FL) signal was measured by Rotor-Gene Q 2plex HRM (QIAGEN N.V., Hilden, Germany).

### Preparation of dsDNA

Weigh an appropriate amount of magnesium chloride hexahydrate and dissolve it in deionized water. Then, add the necessary volume of Tris–HCl (pH 8.0) to prepare a 10× TM buffer, ensuring that the final concentrations of Tris–HCl and magnesium chloride are 10 and 0.5 mM, respectively. Dilute each oligonucleotide solution, whose concentration has been precisely measured, to the required concentration. Transfer an appropriate volume of each diluted oligonucleotide into a 200 μl polymerase chain reaction (PCR) tube, adjusting the final concentration of the activator to 1 μM. The concentration ratio of target strand (TS) to nontarget strand (NTS) is 1:1.1 for all the activators used in the experiment. Next, add 2 μl of 10× TM buffer to the PCR tube. Finally, add enzyme-free deionized water to bring the total volume to 20 μl. Place the PCR tube containing the oligonucleotides into a PCR instrument and set the temperature to 95°C for 5 min and then 4°C for 30 min. The procedure was performed on Rotor-Gene Q 2plex HRM (QIAGEN N.V., Hilden, Germany).

### Explore the effect of activator topology on the Cas12a’s *trans*-cleavage activity

In a 20 μl system, 11 μl of deionized water, 2 μl of 10× NEBuffer r3.1, 2 μl of 1 μM crRNA, and 1 μl of 1 μM Cas12a were combined. The mixture was incubated at 37°C for 15 min. Then, 2 μl of ssDNA reporter (1 μM) and 2 μl of 250 nM dsDNA activator (TS: 250 nM, NTS: 275 nM) was added to reach a final volume of 20 μl. FL intensity was immediately measured at 37°C using the Rotor-Gene Q real-time PCR instrument (QIAGEN N.V., Hilden, Germany). The FAM channel was chosen with a gain value of 7. Each sample was tested in triplicate.

### Explore the effect of activator topology on the Cas12a’s *cis*-cleavage activity

In a 20 μl system, 13 μl of deionized water, 2 μl of 10× NEBuffer r3.1, 2 μl of 1 μM crRNA, and 1 μl of 1 μM Cas12a were combined. The mixture was incubated at 37°C for 15 min. Then, 2 μl of 250 nM dsDNA activator were added to reach a final volume of 20 μl. FL intensity was immediately measured at 37°C using the Rotor-Gene Q real-time PCR instrument (QIAGEN N.V., Hilden, Germany). The HEX channel was chosen with a gain value of 7. Each sample was tested in triplicate.

### Gel electrophoresis

The activator topology was verified using 15% polyacrylamide gel electrophoresis (PAGE) in 0.5× TBE buffer. To prepare the gel, 5 ml of 30% acrylamide/bis-acrylamide (29:1) solution, 3.9 ml of ultrapure water, and 2 ml of 5× TBE buffer were mixed. Then, 100 μl of 10% APS solution and 8 μl of TEMED solution were added to the gel mixture for polymerization. Ten microliters of the prepared activators were premixed with 2 μl of 6× loading buffer and then loaded onto a 15% PAGE gel. Electrophoresis was performed for 60 min at a constant voltage of 120 V. Ten microliters of DNAMarker A (25–500 bp) was used as a molecular weight reference. Gel images were visualized after staining with 4S Red Plus using the fluorescence gel imaging system (Blue Light Gel Imager, G500312, Sangon Biotech Co., Ltd). Pictures were imaged by a mobile phone camera.

### HOCl detection

Acidify the sodium hypochlorite solution with sulfuric acid to form a hypochlorous acid solution. For the “turn on” model, in a 200 μl PCR tube, 2 μl of 250 nM P7-20* activator and 8 μl of hypochlorous acid solution of the corresponding concentration were mixed well. The mixture was incubated at 37°C for 1 h. Then, 1 μl of deionized water, 2 μl of 10× NEBuffer r3.1, 2 μl of 1 μM crRNA, 2 μl of 2 μM invader strand, and 1 μl of 1 μM Cas12a protein were mixed in another PCR tube. The mixture was incubated at 37°C for 15 min. After the incubation, 10 μl of reaction solution was added to the mixture and incubated at 37°C for 30 min. Then, the mixture was placed at 85°C for 5 min to ensure Cas12a inactivation. After the inactivation, 2 μl of 2 μM dsDNA reporter was added in the mixture to reach a final volume of 20 μl. FL intensity was immediately measured at 37°C using the Rotor-Gene Q real-time PCR instrument (QIAGEN N.V., Hilden, Germany). The FAM channel was chosen with a gain value of 7. Each sample was tested in triplicate.

For the “turn off” model, in a 200 μl PCR tube, 2 μl of 250 nM P7-20* activator and 8 μl of hypochlorous acid solution of the corresponding concentration were mixed well. The mixture was incubated at 37°C for 1 h. Then, 3 μl of deionized water, 2 μl of 10× NEBuffer r3.1, 2 μl of 1 μM crRNA, and 1 μl of 1 μM Cas12a protein were mixed in another PCR tube. The mixture was incubated at 37°C for 15 min. After the incubation, we added 10 μl of reaction solution and 2 μl of 2 μM ssDNA reporter to the mixture. FL intensity was immediately measured at 37°C using the Rotor-Gene Q real-time PCR instrument (QIAGEN N.V., Hilden, Germany). The FAM channel was chosen with a gain value of 7. Each sample was tested in triplicate.

### Test recovery rate

Acidify the sodium hypochlorite solution with sulfuric acid to form a hypochlorous acid solution. Add a specific concentration of hypochlorous acid solution to each set of Yangtze River water sample or tap water sample collected to obtain hypochlorous acid samples with a final concentration of 0.25, 2.5, and 250 μM. In a 200 μl PCR tube, 2 μl of 250 nM P7-20* activator and 8 μl of hypochlorous acid of the corresponding concentration were mixed well. The mixture was incubated at 37°C for 1 h. Then, 1 μl of deionized water, 2 μl of 10× NEBuffer r3.1, 2 μl of 1 μM crRNA, 2 μl of 2 μM invader strand, and 1 μl of 1 μM Cas12a protein were mixed in another PCR tube. The mixture was incubated at 37°C for 15 min. After the incubation, 10 μl of reaction solution was added to the mixture and incubated at 37°C for 30 min. Then, the mixture was placed at 85°C for 5 min to ensure Cas12a inactivation. After the inactivation, 2 μl of 2 μM dsDNA reporter was added in the mixture to reach a final volume of 20 μl. FL intensity was immediately measured at 37°C using the Rotor-Gene Q real-time PCR instrument. The FAM channel was chosen with a gain value of 7. Each sample was tested in triplicate. The recovery rate of each group was calculated using the FL signal platform value and linear relationship curve.

## Results and discussion

### The effect of linear activators on Cas12a activity

In this work, we first designed the linear activators with bubble or hairpin conformations. Considering that the TS and NTS of the dsDNA activator play different roles in the CRISPR–Cas12a system, we introduced specific conformations at different positions of the TS and the NTS to explore the effect of the linear activators on the Cas12a enzyme activity, respectively. We selected a characteristic HCV-RNA sequence with a length of 24 nt as the spacer region sequence of crRNA, and a polyT sequence with a length of 15 nt modified with carboxyfluorescein (FAM) and black hole quencher-1 (BHQ-1) at both ends as the reporter of the reaction system based on previous studies [9].

**Figure 1.**
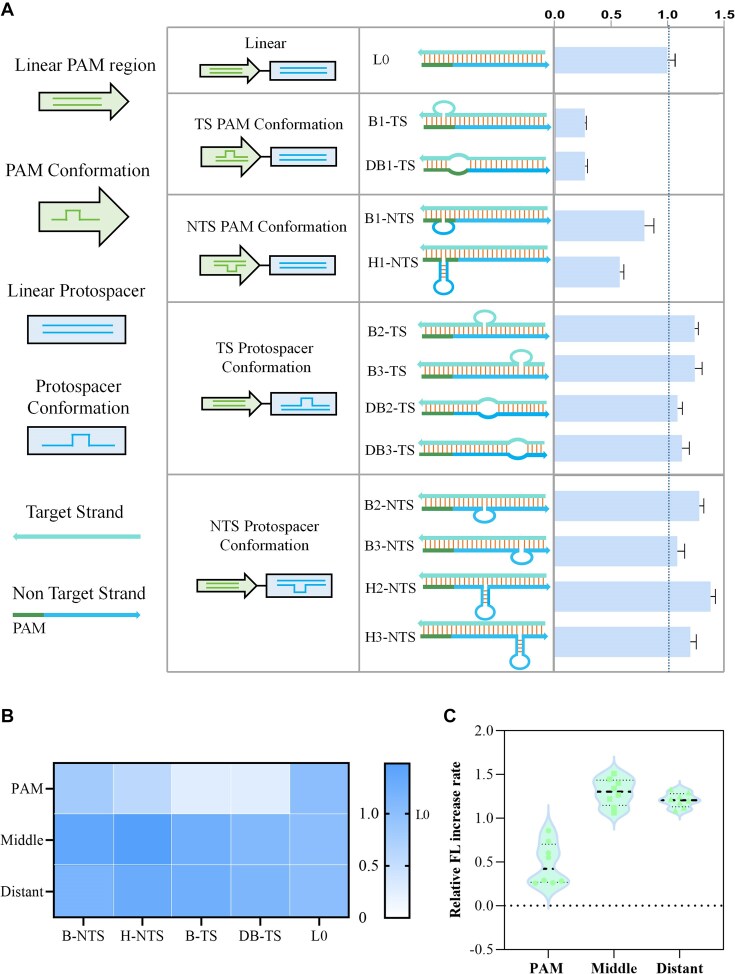
The effect of linear activators on Cas12a enzymatic activity. (**A**) Schematic diagram of design of linear activators, along with the relative FL increase rates of each structure. (**B**) The impact of special structures in the TS and NTS on enzymatic activity. (**C**) The effect of the conformations’ position on Cas12a activity. All relative FL increase rates are normalized to that of L0. Error bars represent the standard deviation from at least three independent replicates.

As shown in Fig. 1A, we designed a bubble-structured activator (bubble, B) with a size of 6 nt, a hairpin-structured activator (hairpin, H) with a 6-bp neck and an 8-nt loop, and a double-bubble-structured activator (DB) with a size of 6 nt. Among them, the activators with NTS conformation not only maintain the complete TS sequence but also have a linear conformation; the activators with TS conformation only maintain the integrity of the sequence. Each structure was designed to be located on TS or NTS in the PAM region, the middle of the protospacer region, or the distal end of the protospacer region of the activator, respectively. When Cas12a is activated, its *trans*-cleavage activity will cleave the reporter to generate a fluorescent signal. Cas12a activity was evaluated by the FL increase rate. We set the relative FL increase rate of the traditional linear dsDNA activators (L0) without a bubble or hairpin as 1.0 to calculate the relative activity of Cas12a on different activators (Fig. 1A).

As shown in Fig. 1B and C, altering the conformation of the PAM region led to varying degrees of activity inhibition. The inhibition was most pronounced when the TS conformation was altered. In contrast, when the structures were modified in the middle or distal of the protospacer, all activators showed an enhancement in enzyme activity, with the NTS conformation changes resulting in better activation. When comparing the effects of hairpin and bubble structures, we found that the hairpin structure, with its more constrained conformation, produced stronger inhibition or enhancement effects. Taken together, for linear activators, the main factors influencing Cas12a enzyme activity in special conformations are the position and degree of freedom. The FL kinetic curves for linear activators are shown in Supplementary Fig. S1.

In the CRISPR–Cas12a system, the PAM recognition is responsible for determining the target (activator) site and facilitating target unwinding [24], while the complementary pairing between the protospacer region and the crRNA spacer region is crucial for generating *trans*-cleavage activity. A change in the PAM region conformation hinders activator recognition and unwinding, thereby inhibiting enzyme activity. In contrast, when the TS sequence remains unchanged, the decrease of freedom in the protospacer region facilitates activator unwinding and enhances enzyme activity. For TS conformations, TS bubbles located in either the middle or distal regions of the protospacer can directly interact with crRNA and accelerate R-loop formation. Structural differences are the primary factor affecting *trans*-cleavage activity, which is why activators with TS bubbles in the central or distal protospacer regions exhibit similar *trans*-cleavage activity. For NTS conformations, the TS remains fully base-paired, and the introduction of redundant structures increases duplex instability, facilitating unwinding and promoting R-loop formation, thereby enhancing *trans*-cleavage activity. However, when a special structure is located at the distal protospacer, the destabilized region is farther from the seed region [25], leading to a slightly slower R-loop formation rate and relatively reduced *trans*-cleavage activity. Besides, we used NUPACK to predict the structures of B3-NTS and B2-NTS. The structure of B3-NTS is more stable than B2-NTS (Supplementary Fig. S2), which may lead to a higher affinity of B2-NTS for crRNA. These factors may collectively contribute to the higher *trans*-cleavage activity of middle protospacer conformations.

### The effect of planar activators on Cas12a activity

**Figure 2.**
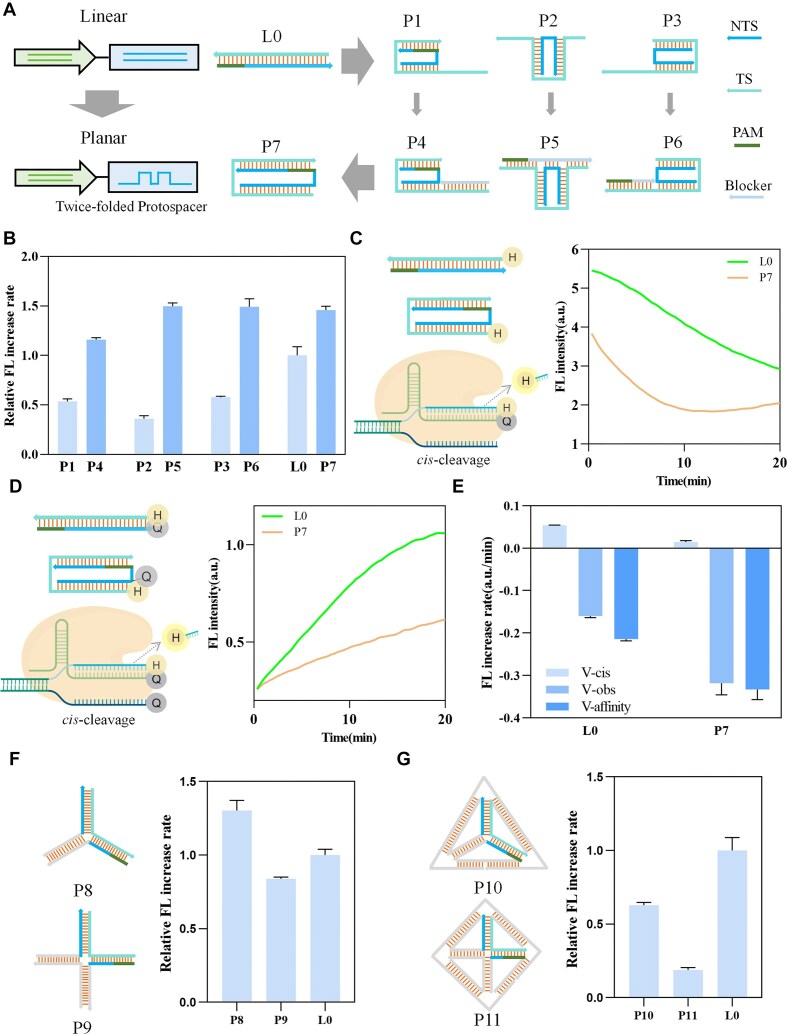
The effect of planar activators on Cas12a enzymatic activity. (**A**) Schematic diagram of design of planar activators. (**B**) The relative FL increase rates of activator P1–P7. (**C**) Schematic diagram of exploring the observed Cas12a *cis-*cleavage activity of L0 and P7, along with their FL kinetic curve. “H” represents fluorophor HEX and “Q” represents quencher BHQ-1. (**D**) Schematic diagram of exploring the Cas12a *cis-*cleavage activity of L0 and P7, along with their FL kinetic curve. (**E**) The V-obs, V-*cis*, and V-affinity of L0 and P7. (**F**) Schematic diagram of design of activators P8 and P9, along with their relative FL increase rates. (**G**) Schematic diagram of design of activators P10 and P11, along with their relative FL increase rates. All relative FL increase rates are normalized to that of L0. Error bars represent the standard deviation from at least three independent replicates.

The linear activators described above contain only one special structure (bubble or hairpin), which occupies a small portion of the activator. Next, we designed a protospacer region with two special structures (twice-folded) by altering the NTS sequence. This design results in a planar structure that maintains the integrity of the TS sequence. Similar to the exploration of the linear structures, we investigated the influence on Cas12a activity by shifting the location of the folded structure (Fig. 2A). As shown in Fig. 2B, activators P1–P3 exhibited varying degrees of inhibition compared to L0. The presence of single-stranded regions in P1–P3 may react with the crRNA through toehold-mediated strand displacement (TMSD) to activate Cas12a with TS ssDNA, leading to decreased enzyme activity.

So, we blocked the single-stranded regions in P1–P3 using a complementary sequence, resulting in activators P4–P6 (Fig. 2A). Additionally, we simplified the structure of P4–P6 to form a symmetric structure, P7, consisting of two strands. The results, shown in Fig. 2B, indicated that all P4–P6 displayed varying degrees of enhancement compared to P1–P3. These results may be attributed to Cas12a's preferential recognition of double-stranded activators. To further explore this, we examined the *trans*-cleavage activity of the double-stranded activator L0 and TS (Supplementary Fig. S3A). The results demonstrate that the FL increase rate of TS is ∼70% of that of L0, thus corroborating our hypothesis. Besides, the results also indicate that all P4–P7 display varying degrees of enhancement compared to L0. We hypothesize that the enhancement is due to the cleavage site of the folded conformation being distant from the RuvC domain of Cas12a. As a result, Cas12a may not efficiently cleave the activator through *cis-*cleavage, and the open active pocket can directly hydrolyze the reporter with *trans*-cleavage (Supplementary Fig. S4).

To validate this hypothesis, we investigated the *cis-*cleavage activity of L0 and P7. As shown in Fig. 2C, the 3' end of the crRNA was modified with the quencher BHQ-1, while the 5' ends of the L0 and P7 TS were modified with the fluorophor HEX. The FL kinetic curves represent the combined process of activator binding to Cas12a ribonucleoprotein (RNP) and the subsequent activation of *cis-*cleavage. The FL increase rate (V-obs) reflects the observed Cas12a *cis-*cleavage activity induced by activator binding. According to the FL curve of P7, during the 0–10 min phase, activator binding to crRNA dominates, and the binding rate is higher than the cleavage rate, causing an overall decline in FL. During the 10–20 min phase, as the binding process nears completion, *cis*-cleavage becomes dominant, leading to an overall increase in FL. So, the first phase was chosen to calculate V-obs. As shown in Fig. 2D, we modified the corresponding positions of the L0 and P7 NTS with BHQ-1, while maintaining the rest of the setup as in Fig. 2C. The BHQ-1 modification on the NTS directly quenched the HEX signal from the TS, masking the FL signal decrease caused by activator binding to Cas12a RNP. Consequently, the FL kinetic curve only displayed the *cis-*cleavage of the activator. The FL increase rate (V-*cis*) represents the rate of Cas12a *cis-*cleavage activity. As shown in Fig. 2E, the V-*cis* of P7 is only 26% of that of L0. The activator binding rate to Cas12a RNP (V-affinity) is the difference between V-obs and V-*cis*. As shown in Fig. 2E, the V-affinity of P7 is ∼1.6 times that of L0. As shown in Fig. 2C, P7 exhibits a stronger binding affinity to the Cas12a RNP compared to L0. Theoretically, this should lead to a faster *cis*-cleavage rate for P7. However, the results in Fig. 2D indicate that P7 has a lower cleavage rate than L0. This suggests that in our experimental design of Fig. 2D, the *cis*-cleavage process remains the dominant factor influencing the overall FL signal, rather than the binding process. These results suggest that activator binding to Cas12a RNP, rather than *cis-*cleavage, is the essential requirement for *trans*-cleavage. In contrast, the presence of *cis-*cleavage may restrict *trans*-cleavage activity. We used this method to validate the *cis*-cleavage activity of the linear activators B3-TS and B3-NTS. The results indicate that the *cis*-cleavage activity of both B3-TS and B3-NTS is weaker than that of L0, which is consistent with our conclusions (Supplementary Fig. S5). Detailed discussion is provided in the Supporting Information.

Next, we designed a three-way junction (P8) and a four-way junction (P9) in the activator, both of which maintained the complete TS sequences. The results, shown in Fig. 2F, reveal that P8 produces an enhancement effect, while P9 exhibites a weaker inhibitory effect compared to L0. Since P8 and P9 can form linear TS via allosterism induced by the Cas12a RNP complex (Supplementary Fig. S3B), their effects on enzyme activity may arise from a combination of two factors: the enhancement of activator unwinding after the allosterism and the inhibitory effect of steric hindrance effect that impedes this transition. The four-way junction in P9 faces greater steric hindrance during the allosterism, resulting in a stronger inhibitory effect than the enhancement of activator unwinding. As a result, P9 shows an overall weak inhibitory effect. In contrast, the three-way junction in P8 leads to an overall enhancement in enzyme activity.

To further reduce the degrees of freedom of P8 and P9, we designed rigid planar triangular and tetrahedral frames and embedded the structures of P8 and P9 into these frames, creating P10 and P11 (Fig. 2G). The results indicate that P10 and P11 exhibit stronger inhibitory effects on enzyme activity compared to P8 and P9. This suggests that the introduction of a rigid structure makes it more difficult for the three-way junctions and four-way junctions to undergo allosterism. Furthermore, these results imply that when the activator maintains the complete TS sequence, the inhibitory effect of planar structures on Cas12a is related to the structural freedom of the activator. The exact mechanism behind this effect requires further investigation. The FL kinetic curves for planar activators are shown in Supplementary Fig. S3C–F.

### The effect of steric activators on Cas12a activity

**Figure 3.**
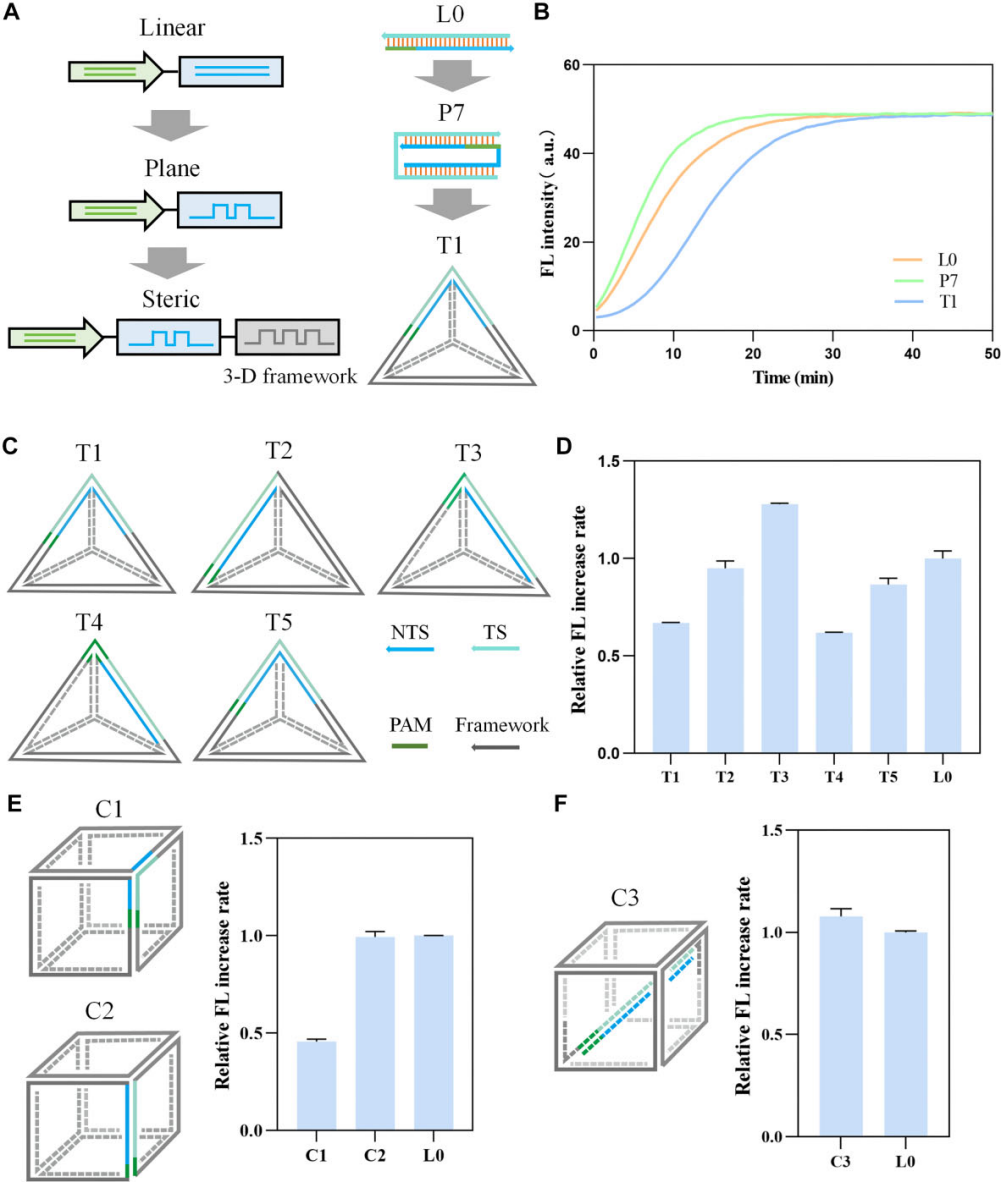
The effect of steric activators on Cas12a enzymatic activity. (**A**) Schematic diagram of design of steric activators. (**B**) FL kinetics of activators T1, P7, and L0. (**C**) Schematic diagram of design of tetrahedral structures T1–T5. (**D**) Relative FL increase rates of activators T1–T5. (**E**) Schematic diagram of design of cubic activators C1 and C2, along with their relative FL increase rates. (**F**) Schematic diagram of design of cubic activator C3, along with its relative FL increase rate. All relative FL increase rates are normalized to that of L0. Error bars represent the standard deviation from at least three independent replicates.

We further explored the effect of steric activators on Cas12a activity. Due to the limited length of the activator sequences, directly constructing steric structures was challenging. Therefore, we constructed a tetrahedral framework (T1) by embedding the activator sequences into the tetrahedron, with the protospacer region located at the apex (Fig. 3A). It is noteworthy that by precisely controlling the chain length, we have induced distinct topological structures between T1 and P10. The AlphaFold 3 prediction results further validate the correct formation of T1 (Supplementary Fig. S6). The tetrahedral structure T1 exhibites some degree of inhibition compared to L0, as well as the planar activator P7 (Fig. 3B).

We created additional tetrahedral activator structures (T2–T5) by embedding the activator sequences at different positions within the tetrahedral framework, ensuring that all maintained the complete TS sequence (Fig. 3C). In these designs, T2 placed the target sequence (PAM and protospacer) at one edge of the tetrahedron, T3 positioned the PAM sequence and protospacer region at two different edges, and T4 positioned the PAM sequence at the apex. The NTS in T1 was split, while T5 was designed to have the same protospacer region location but maintain both the complete TS and NTS sequences. As shown in Fig. 3D, all these five steric activators can activate Cas12a. Compared to L0, T3 exhibited a stronger enhancement effect, while T1, T4, and T5 showed varying degrees of inhibition, and T2 displayed similar activation to L0. These results suggest that the steric framework itself does not significantly affect enzyme activity. However, the rigid folded structure of the activator is the key factor. Both the PAM and protospacer region folded structures tend to reduce enzyme activity, with the folded PAM region producing a stronger inhibitory effect. Surprisingly, when the folded structure is between the PAM and protospacer regions, it leads to a strong activating effect. This may be because the folded structure does not affect the biological function of the PAM and protospacer regions but instead increases activator instability, making it more susceptible to unwinding by crRNA and enhancing *trans*-cleavage activity. Additionally, although a previous study [26] suggests that several nucleotides in the seed region of the spacer are known to interact with the REC lobe of Cas12a, it appears that the middle and distal crRNA spacer regions do not interact as strongly with Cas12a. The PAM region helps Cas12a localize the target sequence, and the conformational transitions of the crRNA spacer region can adapt to the activator structure, facilitating the unwinding process and driving stronger binding. The FL kinetic curves for T1 to T5 are shown in Supplementary Fig. S7A and B.

Following this line of reasoning, we designed a hexahedral activator C1 with the middle of the protospacer region located at the apex of the hexahedron and C2 with the sequences located along one side of the hexahedron (Fig. 3E). The AlphaFold 3 prediction results further validated the correct formation of steric activators (Supplementary Fig. S8). Compared to L0, C1 exhibites a degree of inhibition, while C2 shows a similar activation effect. C1 has stronger inhibitory effects on enzyme activity than T1. This may be because the hexahedral structure induces a greater degree of activator folding than the tetrahedral structure, and these results further support the idea that the crRNA spacer region can shift in response to the activator's conformation.

In addition, we encapsulated the activator sequences within a rigidly structured hexahedral framework to create the C3 activator (Fig. 3F). Surprisingly, C3 shows better activation, which could be due to Cas12a's ability to enter the hexahedral structure and recognize the internal target sequence. Overall, these findings indicate that a steric structure does not completely inhibit enzyme activity when the TS sequence is intact. Instead, the rational design of the activator enhances Cas12a's activity.

**Figure 4.**
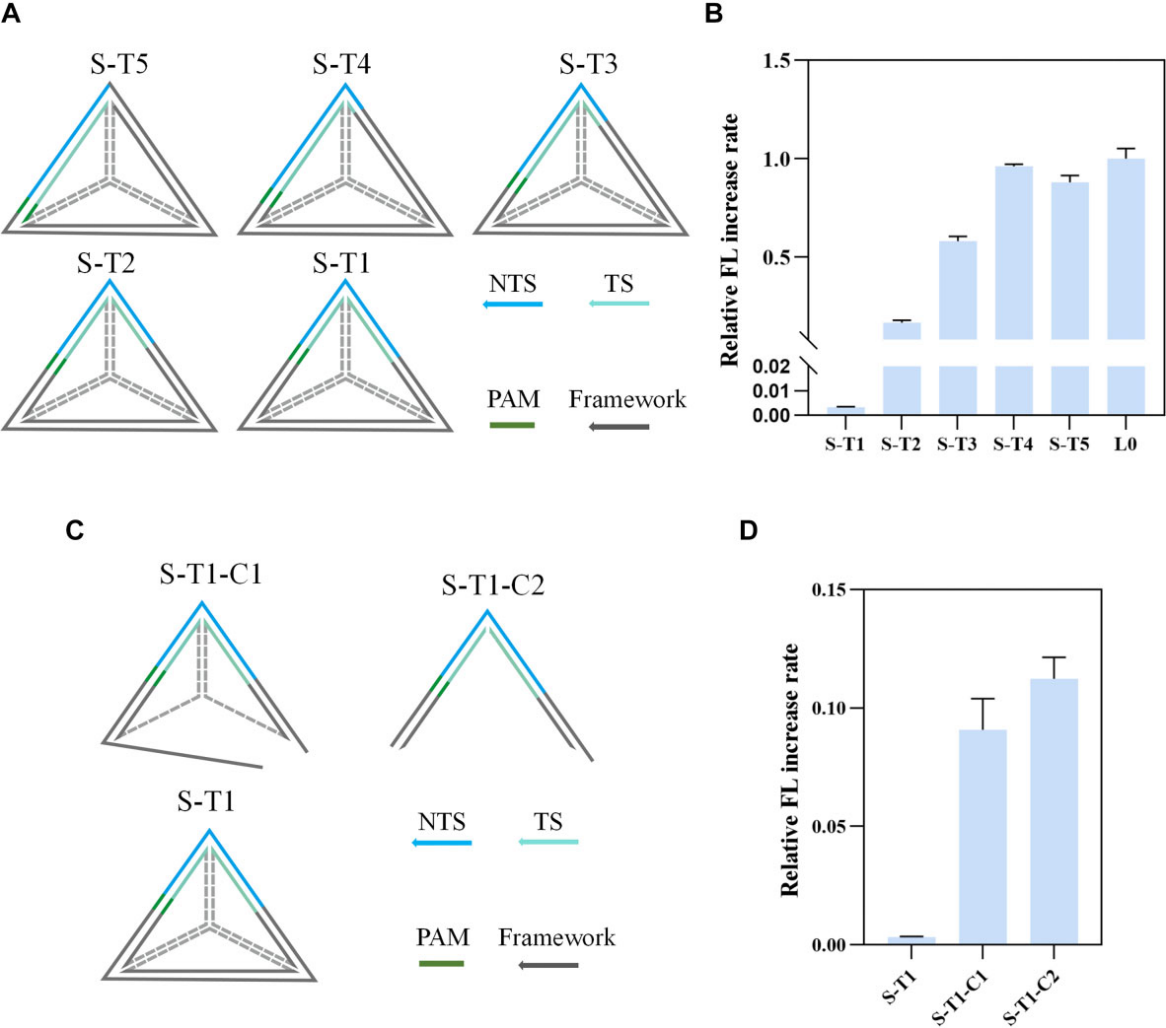
The effect of tetrahedral activators with split TS on Cas12a enzymatic activity. (**A**) Schematic diagram of tetrahedral activators with split TS design. (**B**) Relative FL increase rates of activators S-T1 to S-T5. (**C**) Schematic diagram of control design of control structures S-T1-C1 and S-T1-C2. (**D**) Relative FL increase rates of activators S-T1-C1 and S-T1-C2. All relative fluorescence increase rates are normalized to that of L0. Error bars represent the standard deviation from at least three independent replicates.

We hypothesized that the intact TS structure plays a more significant role than topology in influencing Cas12a's activity. To test this, we designed tetrahedral structures with split TS sequences (Fig. 4A). In these designs, the target sequence of S-T5 was located at one edge of the tetrahedron, and the 5' end of the target sequence was sequentially split by 3, 6, 9, or 12 nt at two edges to create S-T4, S-T3, S-T2, and S-T1. As shown in Fig. 4B and Supplementary Fig. S7C, the closer the split position is to the middle of the protospacer region, the stronger the inhibitory effect. Notably, S-T1 exhibited nearly complete inhibition of Cas12a. Control experiments with the tetrahedral defective structures S-T1-C1 and lacking redundant structures S-T1-C2 (Fig. 4C) showed that the activation rate of these controls was >30 times higher than that of S-T1 in both cases (Fig. 4D and Supplementary Fig. S7D). Our study points out that the folded structure in the middle of the protospacer region and the split TS sequence both exert inhibitory effects (Fig. 3D and Supplementary Fig. S9). However, a split TS activator with a rigid steric structure will enhance mutual inhibition. This property could be leveraged in the development of low-background detection systems with high application potential.

To determine the topology structures' formation and TS leakage, we selected representative activators from linear, planar, and three-dimensional structures for FL electrophoresis analysis (L0, B3-TS, P7, and S-T1). The results in Supplementary Fig. S10 indicate that the synthesized activators do not exhibit TS leakage, confirming their structural stability. Additionally, the activator topology effects are also applicable to different commercial buffers such as NEBuffer r2.1 and NEBuffer 4 (Supplementary Fig. S11).

### The HOCl detection with topological structure allosterism

Next, we used P7 and split TS for HOCl detection. HOCl is a commonly used disinfectant and bleaching agent. However, excessive HOCl in the environment can cause skin damage [27] and neurodegenerative diseases [28], and even pose a life-threatening risk. Recent studies have also linked HOCl to the development of diseases, such as rheumatoid arthritis, lung injury, and cancer [29–31], so it can be used as a diagnostic marker for these conditions. Detecting the level of HOCl plays an important role in ensuring the safety of water resources and controlling disease outbreaks.

**Figure 5.**
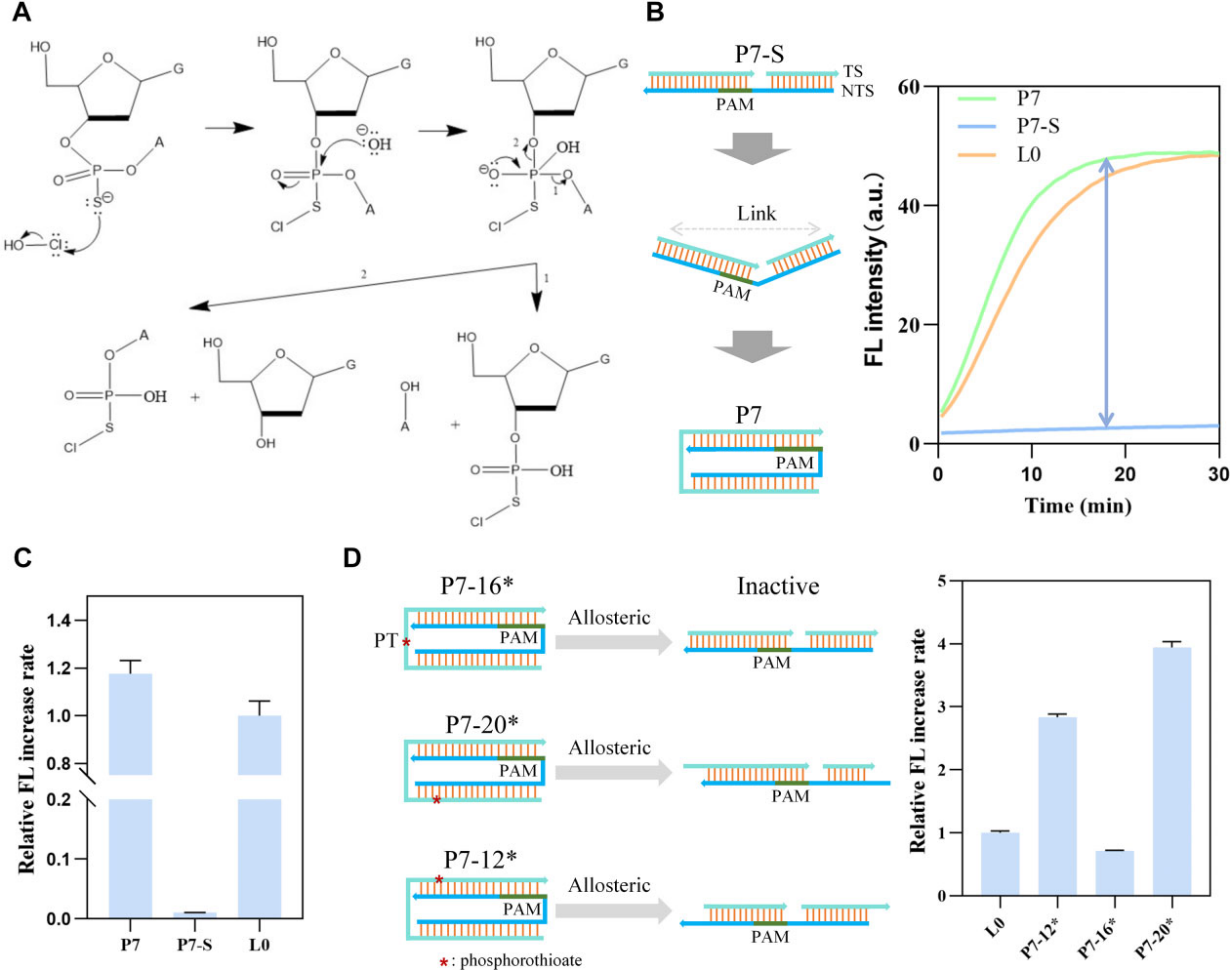
Design of Cas12a switch based on planar activator P7. (**A**) Mechanism of HOCl hydrolyzing the phosphorothioate modifications in the DNA backbone. (**B**) Schematic of the switch based on the planar structure P7, along with the FL kinetic curves for activators P7, P7-S, and L0. (**C**) Relative FL increase rate of activators P7, P7-S, and L0. (**D**) The effect of phosphorothioate sites on enzymatic activity. All relative FL increase rates are normalized to that of L0. Error bars represent the standard deviation from at least three independent replicates.

HOCl can specifically hydrolyze the phosphorothioate modifications in the DNA backbone, leading to DNA cleavage (Fig. 5A). The P7 activator used in Fig. 3C consists of only two strands and contains a segment of ssDNA, making it prone to allosterically regulate. As shown in Fig. 5B, P7-S is the product of cutting off the 16th position of the TS of P7. Due to the traction effect of NTS, the latter half of the activator protospacer region is shifted to the PAM region, preventing the overall sequence from complementary pairing with the crRNA spacer, thereby completely inhibiting Cas12a's enzymatic activity. In contrast, when the 5' and 3' ends of the TS of P7-S are linked, the activity of Cas12a is restored, with P7 activating the enzyme 117-fold more than P7-S (Fig. 5C). So, we modified the single-stranded portion of the TS in P7 by phosphorothioate modification. In the presence of HOCl, the phosphorothioate modification site is specifically cleaved, generating the split structure P7-S, which inhibits Cas12a activity.

Considering that the phosphorothioate sites on the activator may affect enzymatic activity, we designed P7-12*, P7-16*, and P7-20* with phosphorothioate modifications at 12, 16, or 20 positions counting from the PAM (Fig. 5D). P7-16* inhibites Cas12a's activity. The specific reasons still need further investigation. In contrast, P7-12* and P7-20* enhance the Cas12a's activity by 1.8-fold and 2.7-fold compared to L0, respectively. Therefore, P7-20* was used for HOCl detection.

**Figure 6.**
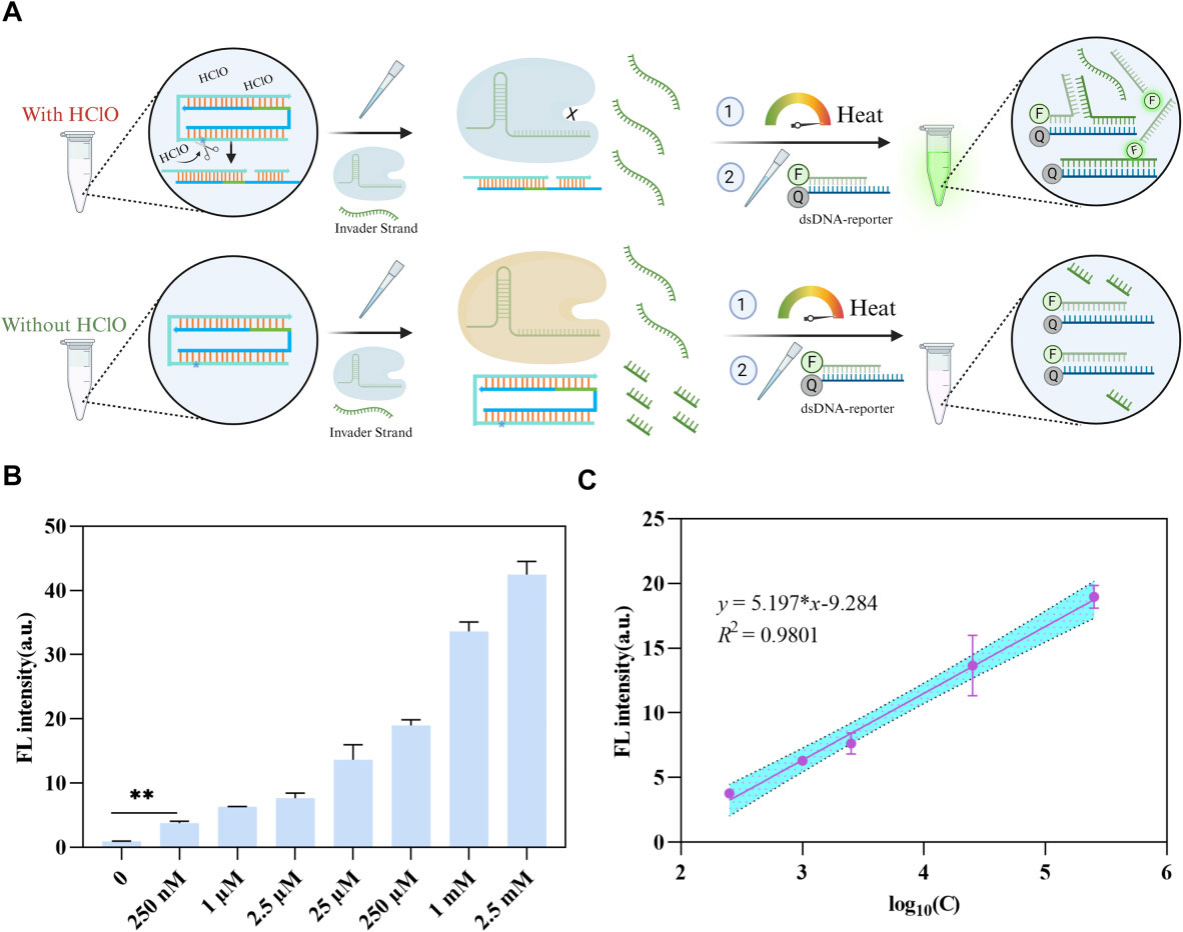
The highly sensitive HOCl detection system is based on Cas12a. (**A**) Schematic diagram for detecting HOCl using the activator P7-20* in the “turn on” model. (**B**) Platform values for detecting HOCl at different concentrations. (**C**) The linear relationship of this detection method. Error bars represent the standard deviation from at least three independent replicates. **P< .01 by Student’s *t-*test.

As shown in Fig. 6A, when HOCl is present in the system, the phosphorothioate site in P7-20* is cleaved, and the activator undergoes allosteric regulation to form a split structure, which inhibits the *trans*-cleavage activity of Cas12a. The invader strand remains intact in the system, while the FAM-modified strand in the dsDNA reporter can be replaced through TMSD to generate a fluorescent signal. In the absence of HOCl, TS in P7-20* remains intact, Cas12a is activated, and the invader strand is cleaved. Thus, TMSD does not occur, and FAM is quenched by BHQ-1 in the dsDNA reporter. As shown in Fig. 6B, the system can detect HOCl concentrations as low as 250 nM. A logarithmic fit of the HOCl concentration to the platform FL signal (Fig. 6C) reveals a strong linear relationship in the concentration range of 250 nM to 250 μM, with the limit of detection of 104 nM, based on the 3*σ*/*k* principle.

To further evaluate the stability of the detection system, three sets of samples were collected from Yangtze River water (Supplementary Fig. S12) or lab tap water. Each set was prepared with standard addition at three different concentrations (high, medium, and low) within the linear range. As shown in Table 1, the recovery rates for all concentration groups of the Yangtze River water samples ranged from 95% to 116%, while those for the tap water samples ranged from 90% to 116%.

**Table 1.** The recovery rate of the HOCl detection method in the “turn on” model

Aqueous matrix	Sample number	Spiked concentration (μM)	Found (mean ± SD) (μM)	Recovery (%)
The Yangtze River water sample	Sample 1	250	248 ±19	99
		2.5	2.38 ±0.28	95
		0.25	0.29 ±0.010	116
	Sample 2	250	275 ±17	110
		2.5	2.74 ±0.65	109
		0.25	0.29 ±0.010	116
	Sample 3	250	236 ±11	95
		2.5	2.55 ±0.82	102
		0.25	0.27 ±0.030	108
The tap water sample	Sample 1	250	254.57 ±16	102
		2.5	2.44 ±0.61	98
		0.25	0.29 ±0.010	116
	Sample 2	250	241 ±17	97
		2.5	2.56 ±0.32	102
		0.25	0.28 ±0.020	112
	Sample 3	250	224 ±14	90
		2.5	2.46 ±1.25	98
		0.25	0.29 ±0.010	116

Alternatively, we directly used the P7-20* activator with ssDNA reporter to construct a Cas12a-based HOCl detection system (Supplementary Fig. S13A). In the presence of HOCl, the phosphorothioate site in P7-20* is cleaved to form a split structure that inhibits Cas12a's *trans*-cleavage activity. The reporter remains intact, and the FL signal is at a lower level according to Förster resonance energy transfer (FRET). In the absence of HOCl, P7-20* remains intact, Cas12a is activated, the reporter is cleaved, and a stronger FL signal is generated. The detection system is capable of detecting HOCl concentrations as low as 250 nM (Supplementary Fig. S13B–D). A logarithmic fit between the HOCl concentration and the plateau FL signal showed a strong linear relationship in the concentration range from 0 nM to 2.5 μM, with a detection limit calculated to be as low as 88 nM, based on the 3*σ*/*k* principle (Supplementary Fig. S13E). The limit of detection(LOD) of this system is 23.1-fold lower than the fluorescent probe method [32] and 11.3-fold lower than the high performance liquid chromatography (HPLC) and UV–Vis spectrometry method [33] (Supplementary Table S2). This enhanced sensitivity is attributed to the powerful sensing ability of the planar activator.

There are also the following two situations that need to be explained. First, due to the distinct reaction mechanisms involved in FL signal generation, “turn on” and “turn off” detection modes used different quantifiable indicators. Due to the rapid strand displacement reaction, the FL signal rise process is difficult to monitor, so the FL plateau intensity was used in the “turn on” mode. Given the multiple-turnover nature of Cas12a *trans*-cleavage activity, the FL increase rate was adopted in the “turn off” mode. Second, the “turn off” mode is simple, quick, and highly sensitive. However, due to the “turn off” principle, the FL signal generated by the system cannot directly indicate the presence of HOCl and must be compared to the FL signal of the negative control. Therefore, we recommend using the previously described “turn on” detection system, which sacrifices some sensitivity but offers a wider linear range and more accurate results, making it more practical.

## Conclusion

In summary, we rationally designed activators with linear, planar, and steric structures based on the principle of DNA base pairing. For the first time, we systematically explored the effect of activator topology on the *trans*-cleavage activity of Cas12a and summarized general principles for designing Cas12a activators. Additionally, our study deepened the understanding of Cas12a's mechanism of activation and introduced a novel concept that activator topology of Cas12a enhances its *trans*-cleavage activity by inhibiting *cis-*cleavage activity. We also developed a novel Cas12a-based HOCl detection system using the concept of allosteric regulation, demonstrating that Cas12a activators with optimized topology offer improved maneuverability and exhibit broad application potential.

## Supplementary Material

gkaf311_Supplemental_File

## Data Availability

The data underlying this article are available in the article and in its online supplementary material.

## Supplementary data

Supplementary data is available at NAR online.
