# Supplementary material for: Exploring the effect of activator topology on CRISPR–Cas12a trans-cleavage activity
Source: Nucleic Acids Res. 2025 Apr 22;53(8):gkaf311. doi: 10.1093/nar/gkaf311 (PMC12014286; doi:10.1093/nar/gkaf311)
Supplement: gkaf311_Supplemental_File [file gkaf311_supplemental_file.pdf]

Supporting Information

Exploring the Effect of Activator Topology on CRISPR-Cas12a Trans-Cleavage Activity

Zixuan Zhu[1,†], Xiaolong Li[1,†], Lin Ding[1] and Tongbo Wu[1,*]

1 School of Pharmacy, Tongji Medical College, Huazhong University of Science and Technology, Wuhan, 430030, China

* To whom correspondence should be addressed. Tel: +86 27 83692754; Fax:+86 27 83692754 ; Email: wutongbo@hust.edu.cn

† The first two authors should be regarded as Joint First Authors.

Discussion

It is worth noting that our conclusion may initially seem contradictory to the findings of the previous study (1). However, we believe our study provides a complementary perspective that extends the current understanding of the relationship between *cis*- and *trans*-cleavage.

The study on Molecular Cell primarily utilized full-length double-stranded DNA (dsDNA) activators and concluded that the dissociation of *cis*-cleavage products clears the catalytic site and initiates *trans*-cleavage. However, by modifying the backbone of the dsDNA activators, the authors also demonstrated that incomplete *cis*-cleavage impairs *trans*-cleavage, suggesting that the relationship between these two activities is more complex than initially thought.

Further supporting evidence comes from Doudna et al. (2), who used gel electrophoresis to study the effect of short dsDNA activators on *trans*-cleavage. Their results showed strong *trans*-cleavage activity in dsDNA substrates of 10–20 bp without detectable *cis*-cleavage, indicating that *trans*-cleavage can occur independently of *cis*-cleavage under certain conditions. Additionally, Segerink et al. (3) used fluorescence-based methods to investigate activator length and found that a 20-bp activator induced stronger *trans*-cleavage activity than a full-length 24-bp activator. The authors suggested that spatial hindrance from the cleavage site might slow down trans-cleavage activity, while shorter activators do not interfere with the RuvC catalytic site, allowing direct ssDNA cleavage without requiring complete dissociation of the *cis*-cleavage product. More recently, Sanghwa Lee et al. (4) proposed using Cas12a variants to suppress spatial hindrance from *cis*-cleavage products and enhance *trans*-cleavage activity. Their study demonstrated that a 19-nt single-stranded activator outperformed the full-length TS, and a Cas12a variant with reduced affinity for the cleaved DNA end increased trans-cleavage activity by 5.8-fold.

Building on these findings, our study introduces a novel approach—modulating the topological structure of dsDNA activators to mitigate the inhibitory effect of *cis*-cleavage on *trans*-cleavage activity. Our results support the idea that, under specific conditions, *cis*-cleavage products can impose steric hindrance that limits *trans*-cleavage efficiency.

Table S1 Oligonucleotide sequences used in this work

Name	DNA sequence (5'-3')
**Reporter used in the experiments**	
ss-reporter	FAM-TTTTTTTTTTTTTTT-BHQ1
ds-reporter-L1	FAM -GGTTCGCTATAGAGTGCTTAT
ds-reporter-L2	AGTCTGATAAGCACTCTATAGCGAACC -BHQ1
**RNA sequences used in the experiment**	
crRNA-HCV-24	UAAUUUCUACUAAGUGUAGAUCUCAGGGCGGACGAGGUUUAGGAU
crRNA-BHQ-1	UAAUUUCUACUAAGUGUAGAUCUCAGGGCGGACGAGGUUUAGGAU-BHQ-1
**DNA sequences used in the experiment**	
L0-TS	ATCCTAAACCTCGTCCGCCCTGAGTAAATGAA
L0-NTS	TTCATTTACTCAGGGCGGACGAGGTTTAGGAT
B1-TS-L2	TTCACAGGGCGGACGAGGTTTAGGAT
DB1-TS-L2	TTCAGGGCAGCAGGGCGGACGAGGTTTAGGAT
B1-NTS-L2	TTCATTATGTGCTACTCAGGGCGGACGAGGTTTAGGAT
H1-NTS-L2	TTCATTTTTGGGTGCTCAGGCCCAAATACTCAGGGCGGACGAGGTTTAGGAT
B2-TS-L2	TTCATTTACTCAGGGAGGTTTAGGAT
B3-TS-L2	TTCATTTACTCAGGGCGGACGAGGAT
DB2-TS-L2	TTCATTTACTCAGGTATTCAGAGGTTTAGGAT
DB3-TS-L2	TTCATTTACTCAGGGCGGACGATTGGGCGGAT
B2-NTS-L2	TTCATTTACTCAGGGCATGTGCGGACGAGGTTTAGGAT
B3-NTS-L2	TTCATTTACTCAGGGCGGACGAGGTTATGTGCTAGGAT
H2-NTS-L2	TTCATTTACTCAGGGCTTTGGGTGCTCAGGCCCAAAGGACGAGGTTTAGGAT
H3-NTS-L2	TTCATTTACTCAGGGCGGACGAGGTTTTTGGGTGCTCAGGCCCAAATAGGAT
P1-L2	GGCGGACGATTCATTTA
P2-L2	GAGGTTTATACTCAGGG
P3-L2	TTTAGGATCAGGGCGGA
P4-L3	AGGTTTAGGAT
P5-L3	TTCATTTAGGAT
P6-L3	TTCATTTACTC
P7-L2	GACGAGGTTTAGGATCGTTCATTTACTCAGGG
P8-L2	TTCATTTACTCAGGGCGGACATAAAGCGGTTA
P8-L3	TAACCGCTTTATGAGGTTTAGGAT
P9-L2	TTCATTTACTCAGGGCGGACATAAAGCGGTTA
P9-L3	TAACCGCTTTATGTTGGTGCGCTC
P9-L4	GAGCGCACCAACGAGGTTTAGGAT
P10-L1	ATCCTAAACCTCGTCCGCCCTGAGTAAAAAAATACCCAAGCTACTCAACAG
P10-L2	GATGAGCAAACATGACGAGGTTTAGGATAGCGAACTACTGTCCCCATGTTTG
P10-L3	GTTGGTGCGCTCTTTACTCAGGGCGGCATGTTTGCTCATCCTCATCGGGATA
P10-L4	CTTGGGTATTTTGGAGCGCACCAACTATCCCGATGAGGCAAACATGGGGACA<br>GTAGTTCGCTGACTGTTGAGTAG
P11-L1	ATCCTAAACCTCGTCCGCCCTGAGTAAAAAAATACCCAAGCTACTCAA
P11-L2	AGCGAACTACTGTCCCCATGGATGAGCAAGAAACATGAGGTTTAGGAT
P11-L3	GGGATAAAGCGGTTAGAATGGAGCGCACCAACATGTTTCTTGCTCATC
P11-L4	TTTACTCAGGGCGGACGTTGGTGCGCTCCATGTTTGCTCATCCTCATC
P11-L5	GTAGTTCGCTTTGAGTAGCTTGGGTATTTTGATGAGGATGAGCAAACATGCATT<br>CTAACCGCTTTATCCCCATGGGGACA

P7-L2-BHQ-1	GACGAGGTTTAGGATCGT-BHQ-1-TCATTTACTCAGGG
TS-HEX	HEX- ATCCTAAACCTCGTCCGCCCTGAGTAAATGAA
NTS-BHQ-1	TTCATTTACTCAGGGCGGACGAGGTTTAGGAT-BHQ-1
B3-TS-L2-BHQ-1	TTCATTTACTCAGGGCGGACGAGGAT-BHQ-1
T1-L1	GGAGCTCACACTCTACTCAACAGTAGCGAACTACTGGACCCGTCCTTCAATCCTAAACCTCGTCCGCCCTGAGTAAA
T1-L2	GTTGGTGCGCTCAAAATACCCAAGAGTGTGAGCTCCTTTACTCAGGGCGGACGTCCTGTCAGCATTCTAACCGCTT
T1-L3	CTTGGGTATTTTGAGCGCACCAACTATCCCGATGAGCAAACATGGGGACAGTAGTTCGCTACTGTTGAGTAG
T1-L4	TCCCCATGTTTGCTCATCGGGATAAAGCGGTTAGAATGCTGACAGGACGAGGTTTAGGATTGAAGGACGGGTC
T2-L1	GGAGCTCACACTCTACTCAACAGTAGCGAACTACTGGACCCGTCCTTCATTTACTCAGGGCGGACGAGGTTTAGGAT
T2-L2	GTTGGTGCGCTCAAAATACCCAAGACTGTTGAGTAGAGTGTGAGCTCCGTCCTGTCAGCATTCTAACCGCTT
T2-L3	CTTGGGTATTTTGAGCGCACCAACTATCCCGATGAGCAAACATGGGGAGAAGGACGGGTCCAGTAGTTCGCT
T2-L4	TCCCCATGTTTGCTCATCGGGATAAAGCGGTTAGAATGCTGACAGGACATCCTAAACCTCGTCCGCCCTGAGTAAAT
T3-L1	AGCGAACTACTGCAATCCTAAACCTCGTCCGCCCTGAGTAAAGGAGCTCACACTACCCGTCCTTTACTCAACAGT
T3-L2	GTTGGTGCGCTCAAAATACCCAAAAGGACGGGTAGTGTGAGCTCCTTTAGTCCTGTCAGCATTCTAACCGCTT
T3-L3	CTTGGGTATTTTGAGCGCACCAACTATCCCGATGAGCAAACATGGGGACAGTAGTTCGCTACTGTTGAGTAG
T3-L4	TCCCCATGTTTGCTCATCGGGATAAAGCGGTTAGAATGCTGACAGGACCTCAGGGCGGACGAGGTTTAGGATTG
T4-L1	AGCGAACTACTGATCCTAAACCTCGTCCGCCCTGAGTAAAGGAGCTCACACTACCCGTCCTTCATACTCAACAGT
T4-L2	GTTGGTGCGCTCAAAATACCCAATGAAGGACGGGTAGTGTGAGCTCCTTGTCCTGTCAGCATTCTAACCGCTT
T4-L3	CTTGGGTATTTTGAGCGCACCAACTATCCCGATGAGCAAACATGGGGACAGTAGTTCGCTACTGTTGAGTAG
T4-L4	TCCCCATGTTTGCTCATCGGGATAAAGCGGTTAGAATGCTGACAGGACTACTCAGGGCGGACGAGGTTTAGGAT
T5-L1	TACTCAACAGTAGCGAACTACTGGACCCGTCCTTCATTTACTCAGGGCGGACGAGGTTTAGGATGGAGCTCACACTC
T5-L2	CTTGGGTATTTTGAGCGCACCAACTATCCCGATGAGCAAACATGGGGACAGTAGTTCGCTACTGTTGAGTAG
T5-L3	GTCCTGTCAGCATTCTAACCGCTTGTTGGTGCGCTCAAAATACCCAAGAGTGTGAGCTCC
T5-L4	TGAAGGACGGGTCTCCCCATGTTTGCTCATCGGGATAAAGCGGTTAGAATGCTGACAGGAC
T5-L5	ATCCTAAACCTCGTCCGCCCTGAGTAAA
C1-L1	ACGAGGTTTAGGATCTTCCTTCCTTCCTTCCTTCGCAAGTGTGGAACTGCTCGCACGCACACCTTCCTTCCTTCCTTCCTTCTTTACTCAGGGCGG
C1-L2	AGTTCCACACTTGCCTTCCTTCCTTCCTTCCTTCCTGACCAGTGAACTGCTCTCAGCAAACCCTTCCTTCCTTCCTTCCTTCGTGTGCGTGCGAGC

C1-L3	AGTTCACTGGTCAGCTTCCTTCCTTCCTTCCTTCCTACCGATAGAACTGCTCCCGCTAGTTGCTTCCTTCCTTCCTTCCTTCGGTTTGCTGAGAGC
C1-L4	AGTTCTATCGGTAGCTTCCTTCCTTCCTTCCTTCATCCTAAACCTCGTCCGCCCTGAGTAAACTTCCTTCCTTCCTTCCTTCCAACTAGCGGGAGC
C1-X	GAAGGAAGGAAGGAAGGAAG
C2-L1	TCAGCCGCTAGTCTTCCTTCATCCTAAACCTCGCAAGTGTGGAACTGCTCGCACGCACACCTTCCTTCATCCTAAACCTCGTCCGCCCTGAGTAAA
C2-L2	AGTTCCACACTTGCCTTCCTTCATCCTAAACCTCCTGACCAGTGAACTGCTCTCAGCAAACCCTTCCTTCATCCTAAACCTCGTGTGCGTGCGAGC
C2-L3	AGTTCACTGGTCAGCTTCCTTCATCCTAAACCTCCTACCGATAGAACTGCTCCCGCTAGTTGCTTCCTTCATCCTAAACCTCGGTTTGCTGAGAGC
C2-L4	AGTTCTATCGGTAGCTTCCTTCATCCTAAACCTCACTAGCGGCTGATTTACTCAGGGCGGACCTTCCTTCATCCTAAACCTCCAACTAGCGGGAGC
C2-X	GAGGTTTAGGATGAAGGAAG
C3-L1	CTTCCTTCCTTCCTTCCTTCGGAACTGCTCGCACGCACACGGGCCTTCCTTCCTTCCTTCCTTCGCCCTTTGACAG
C3-L2	GCGAGCAGTTCCCTTCCTTCCTTCCTTCCTTCTGAACTGCTCTCAGCAAACCGGGCCTTCCTTCCTTCCTTCCTTCGCCCGTGTGCGT
C3-L3	GAGAGCAGTTCACTTCCTTCCTTCCTTCCTTCAGAACTGCTCCCGCTAGTTGGGGCCTTCCTTCCTTCCTTCCTTC
C3-L4	GGGAGCAGTTCTCTTCCTTCCTTCCTTCCTTCGCGCACCAACGCCTGTCAAAGGGCCTTCCTTCCTTCCTTCCTTCGCCCCAACTAGC
C3-X	GAAGGAAGGAAGGAAGGAAGGAAGGAAGGAAGGAAGGAAGGAAGGAAGGAAGGAAGGAAGGAAGGAAGGAAGGAAGGAAG
C3-NTS	GCGTTGGTGCGCTTTTTCATTTACTCAGGGCGGACGAGGTTTAGGATTTTGCCCGGTTTGCT
C3-TS	ATCCTAAACCTCGTCCGCCCTGAGTAAATGAA
S-T1-L1	GGAGCTCACACTCTACTCAACAGTAGCGAACTACTGGACCCGTCCTTCATTTACTCAGGGCGGACGAGGTTTAGGAT
S-T1-L2	GTTGGTGCGCTCAAAATACCCAAGAGTGTGAGCTCCATCCTAAACCTCGTCCTGTCAGCATTCTAACCGCTT
S-T1-L3	CTTGGGTATTTTGAGCGCACCAACTATCCCGATGAGCAAACATGGGGACAGTAGTTCGCTACTGTTGAGTAG
S-T1-L4	TCCCCATGTTTGCTCATCGGGATAAAGCGGTTAGAATGCTGACAGGACGTCCGCCCTGAGTAAATGAAGGACGGGTC
S-T1-L1-HEX	HEX-GGAGCTCACACTCTACTCAACAGTAGCGAACTACTGGACCCGTCCTTCATTTACTCAGGGCGGACGAGGTTTAGGAT
S-T2-L1	GGAGCTCACACTCTACTCAACAGTAGCGAACTACTGGACCCGTCCTTCATTTACTCAGGGCGGACGAGGTTTAGGAT
S-T2-L2	GTTGGTGCGCTCAAAATACCCAAGTAGAGTGTGAGCTCCATCCTAAACGTCCTGTCAGCATTCTAACCGCTT
S-T2-L3	CTTGGGTATTTTGAGCGCACCAACTATCCCGATGAGCAAACATGGGGAGTCCAGTAGTTCGCTACTGTTGAG
S-T2-L4	TCCCCATGTTTGCTCATCGGGATAAAGCGGTTAGAATGCTGACAGGACCTCGTCCGCCCTGAGTAAATGAAGGACGG
S-T3-L1	GGAGCTCACACTCTACTCAACAGTAGCGAACTACTGGACCCGTCCTTCATTTACTCAGGGCGGACGAGGTTTAGGAT
S-T3-L2	GTTGGTGCGCTCAAAATACCCAAGGAGTAGAGTGTGAGCTCCATCCTAGTCCTGTCAGCATTCTAACCGCTT

S-T3-L3	CTTGGGTATTTTGAGCGCACCAACTATCCCGATGAGCAAACATGGGGACGGGT CCAGTAGTTCGCTACTGTT
S-T3-L4	TCCCCATGTTTGCTCATCGGGATAAAGCGGTTAGAATGCTGACAGGACAACCT CGTCCGCCCTGAGTAAATGAAGGA
S-T4-L1	GGAGCTCACACTCTACTCAACAGTAGCGAACTACTGGACCCGTCCTTCATTTA CTCAGGGCGGACGAGGTTTAGGAT
S-T4-L2	GTTGGTGCGCTCAAAATACCCAAGGTTGAGTAGAGTGTGAGCTCCATCGTCCT GTCAGCATTCTAACCGCTT
S-T4-L3	CTTGGGTATTTTGAGCGCACCAACTATCCCGATGAGCAAACATGGGGAGGAC GGGTCCAGTAGTTCGCTACT
S-T4-L4	TCCCCATGTTTGCTCATCGGGATAAAGCGGTTAGAATGCTGACAGGACCTAAA CCTCGTCCGCCCTGAGTAAATGAA
S-T1-C1-L1	GACCCGTCCTTCATTTACTCAGGGCGGACGAGGTTTAGGATGGAGCTCACACT C
S-T1-C1-L2	GTTGGTGCGCTCAAAATACCCAAGAGTGTGAGCTCCATCCTAAACCTC
S-T1-C1-L3	GTCCGCCCTGAGTAAATGAAGGACGGGTCTCCCCATGTTTGCTCATCGGGATA
P7-S-L1	ATCCTAAACCTCGTCC
P7-S-L2	GCCCTGAGTAAATGAA
P7-S-L3	GACGAGGTTTAGGATCGTTCATTTACTCAGGG
TS-12*	ATCCTAAACCTC*GTCCGCCCTGAGTAAATGAA
TS-16*	ATCCTAAACCTCGTCC*GCCCTGAGTAAATGAA
TS-20*	ATCCTAAACCTCGTCCGCCC*TGAGTAAATGAA
Invader Strand	GGTTCGCTATAGAGTGCTTATCAGACT

Notes: All L1 sequences without special indication are TS sequences; * indicates phosphorothioate site

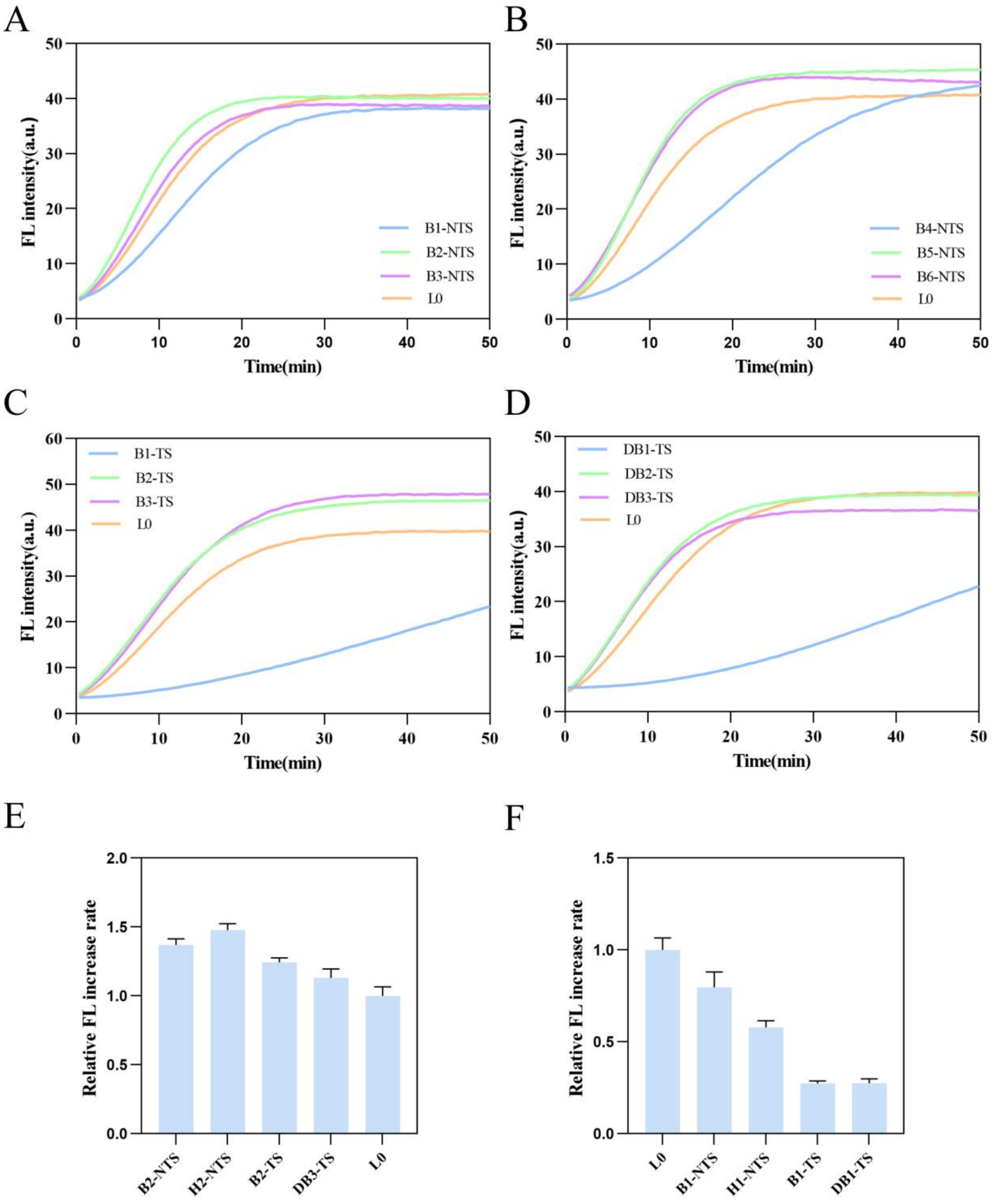

Figure S1 (A) The fluorescence kinetic curves for B1-NTS, B2-NTS, B3-NTS and L0. (B) The fluorescence kinetic curves for B4-NTS, B5-NTS, B6-NTS and L0. (C) The fluorescence kinetic curves for B1-TS, B2-TS, B3-TS and L0. (D) The fluorescence kinetic curves for DB1-TS, DB2-TS, DB3-TS and L0. (E) The relative fluorescence increase rates of all linear structures producing inhibition. (F) The relative fluorescence increase rates of all linear structures producing enhancement. All relative fluorescence increase rates are normalized to that of L0. Error bars represent the standard deviation from at least three independent replicates.

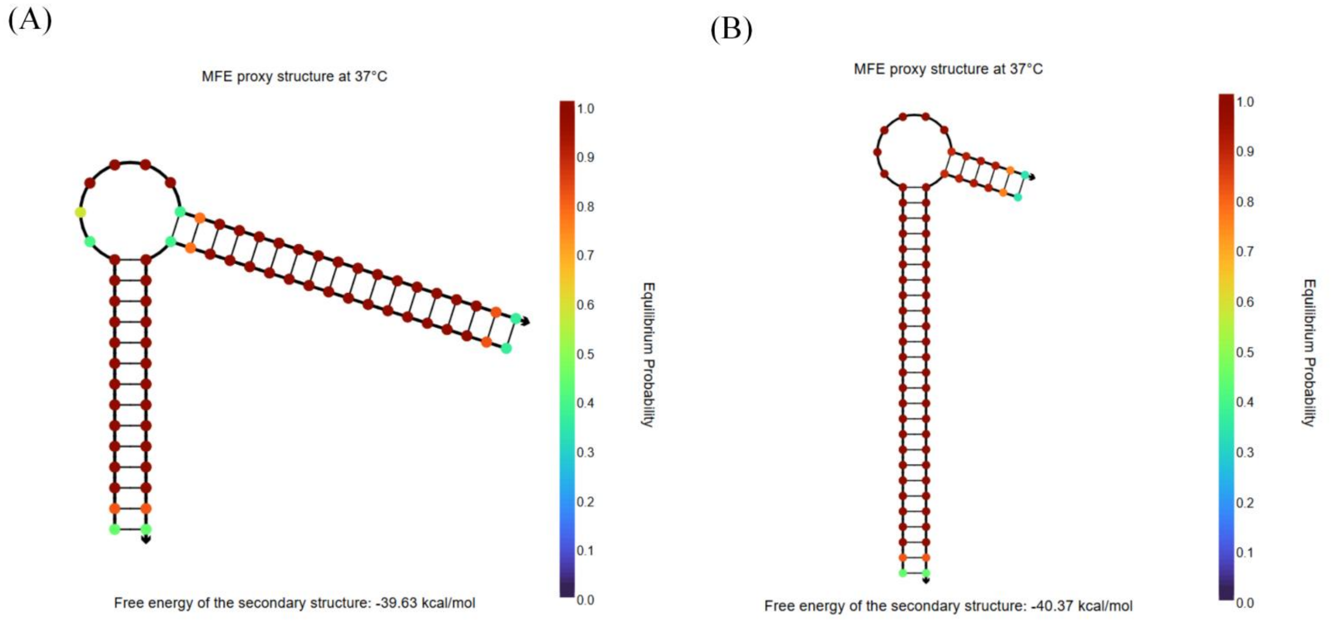

Figure S2 (A) NUPACK prediction of the Gibbs free energy of B2-NTS. (B) NUPACK prediction of the Gibbs free energy of B3-NTS.

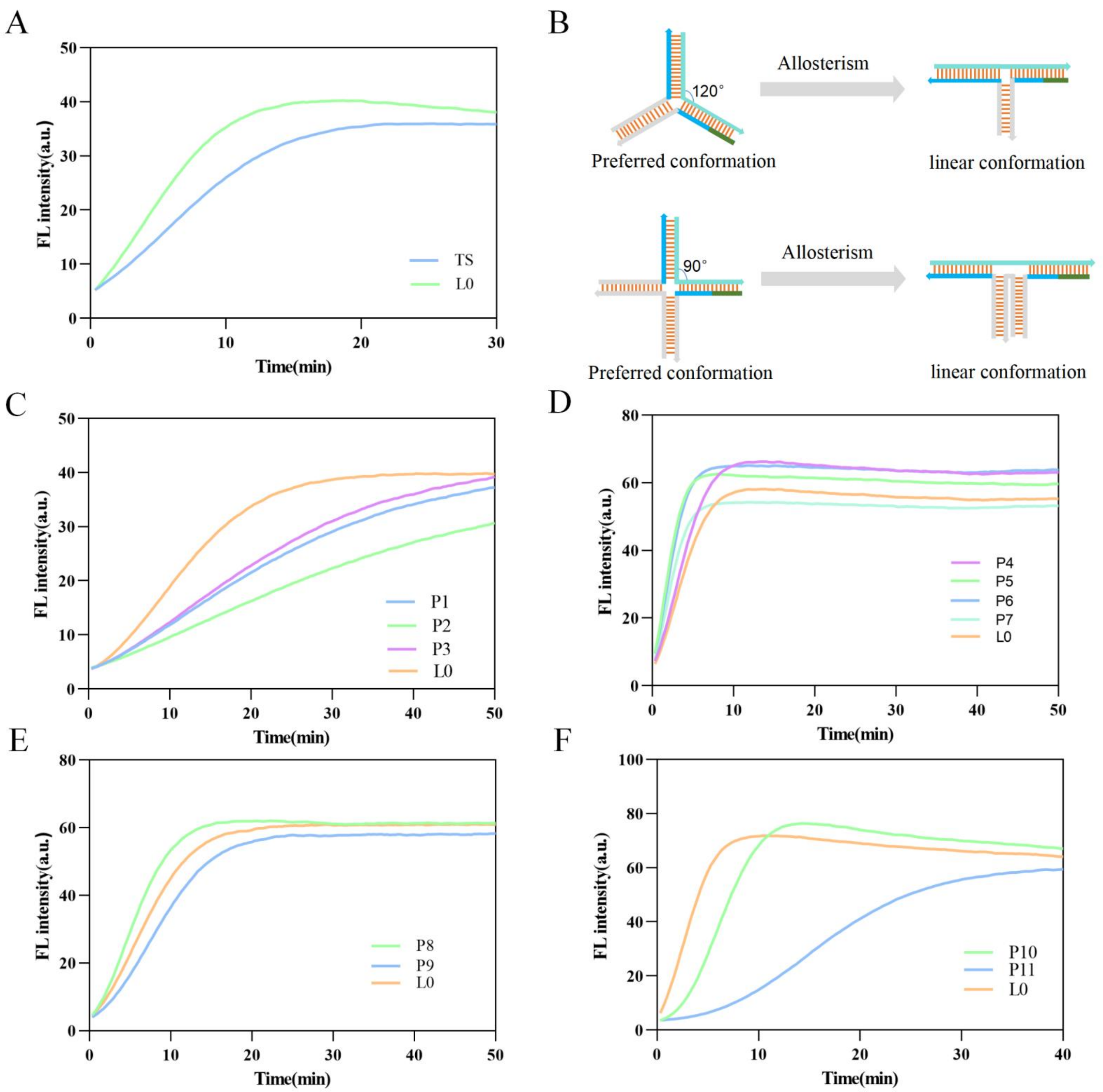

Figure S3 (A) The fluorescence kinetic curves for TS and L0. (B) Schematic diagram of allosterism of P8 and P9.(C) The fluorescence kinetic curves for P1-P3 and L0. (D) The fluorescence kinetic curves for P4-P7 and L0. (E) The fluorescence kinetic curves for P8, P9 and L0. (F) The fluorescence kinetic curves for P10, P11 and L0.

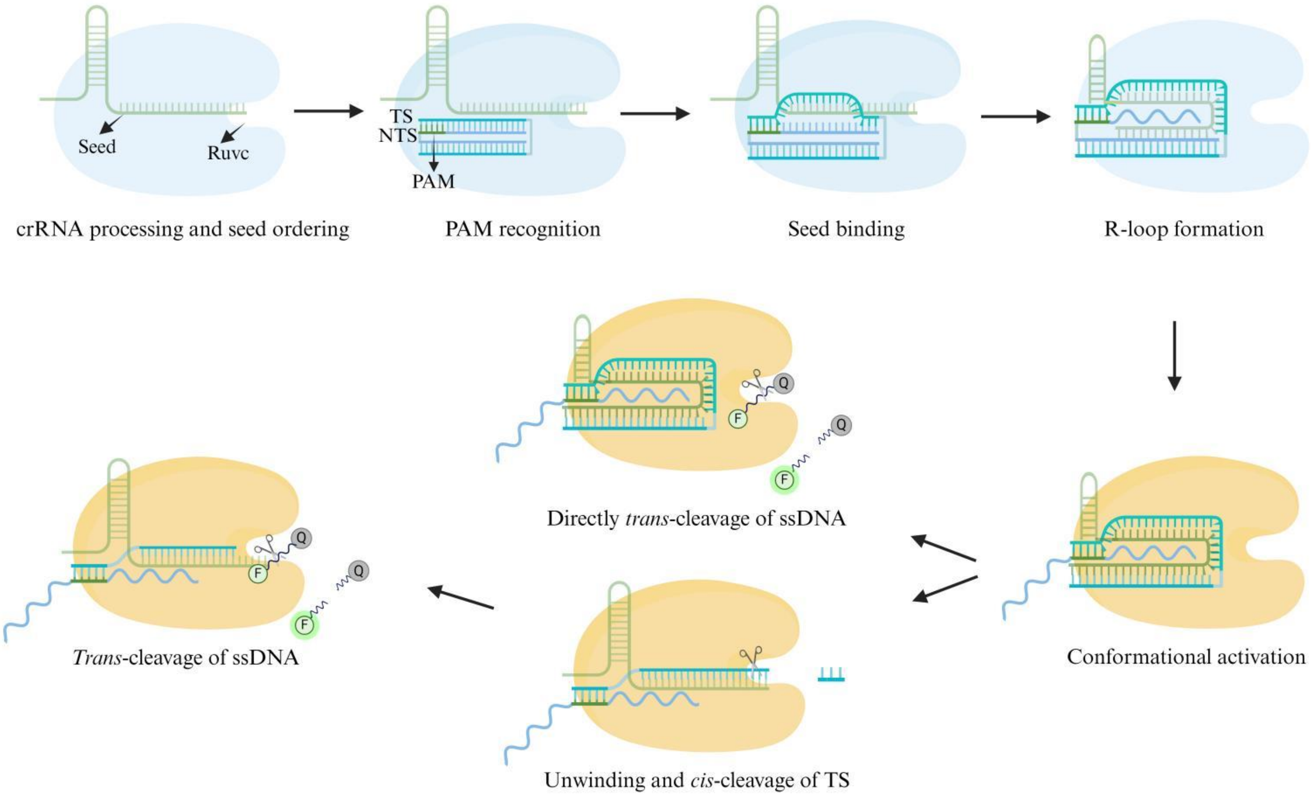

Figure S4 Schematic diagram of topological substrate P7 activating Cas12a.

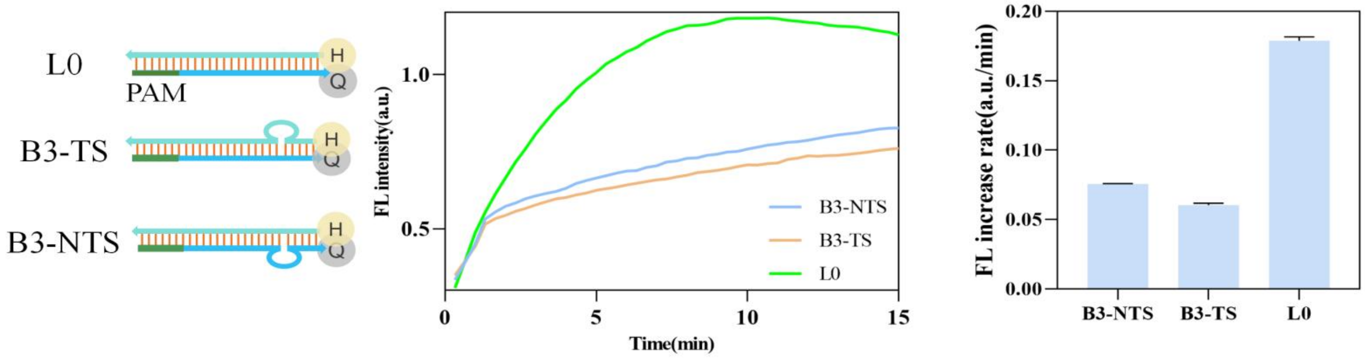

Figure S5 Schematic diagrams of testing the effect of PAM-distal bubble structures on *cis*-cleavage activity along with their fluorescence kinetic curves and relative fluorescence increase rates. All relative fluorescence increase rates are normalized to that of L0. Error bars represent the standard deviation from at least three independent replicates.

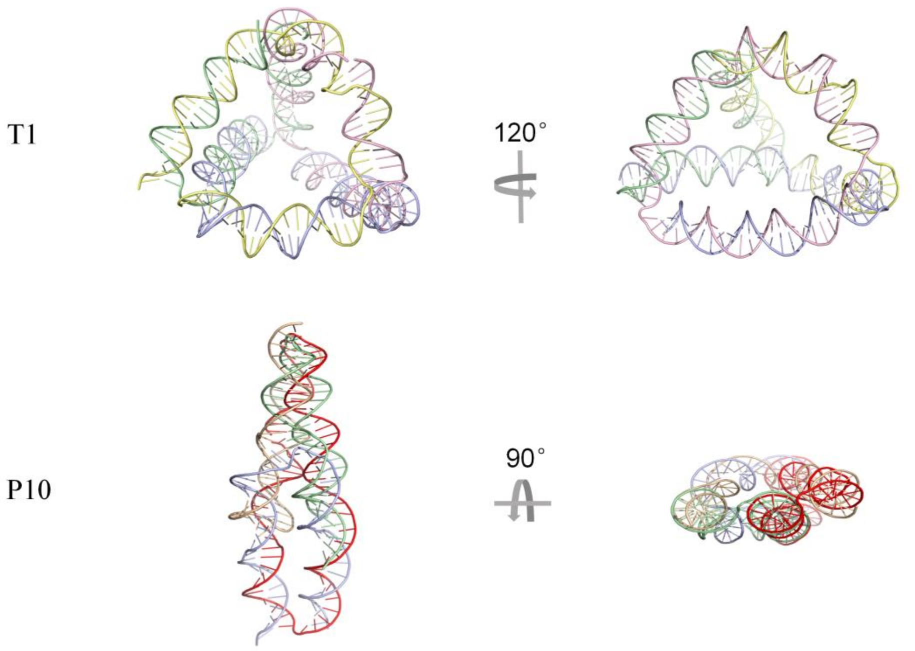

Figure S6 Simulation images of substrates P10 and T1 using AlphaFold 3.

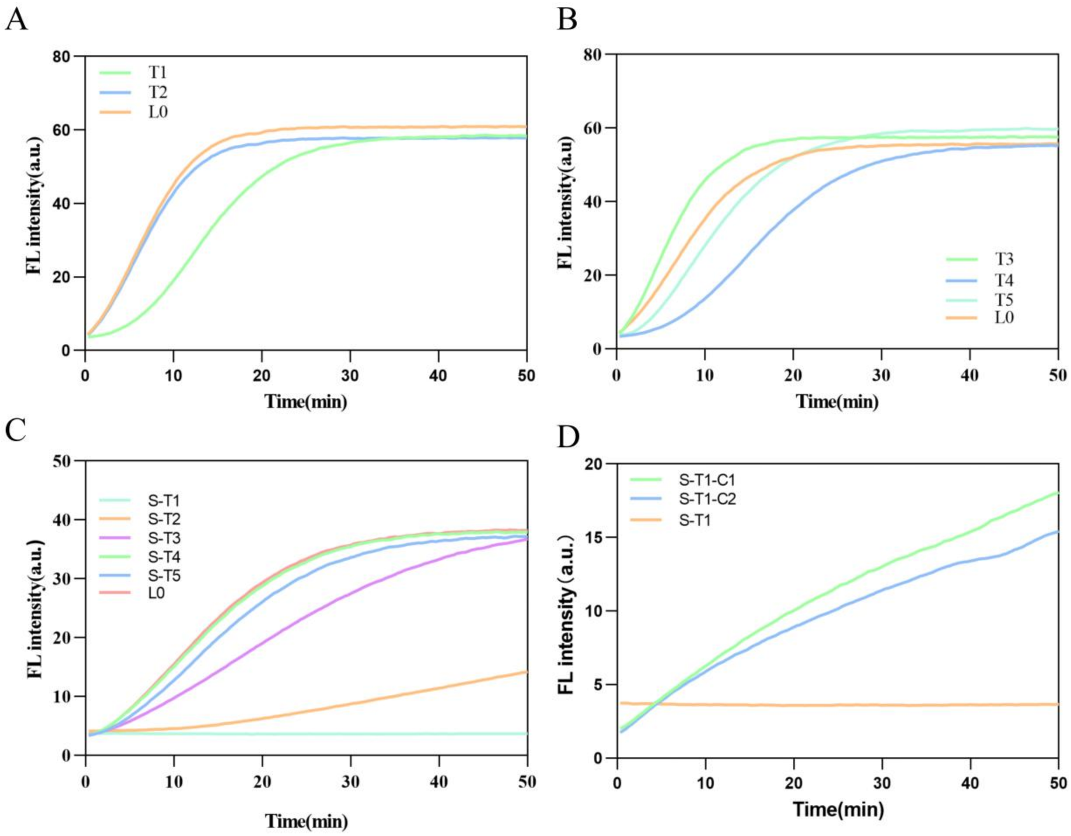

Figure S7 (A) The fluorescence kinetic curves for T1, T2 and L0. (B) The fluorescence kinetic curves for T3, T4, T5 and L0. (C) The fluorescence kinetic curves for S-T1 to S-T5 and L0. (D) The fluorescence kinetic curves for S-T1-C1, S-T1-C2 and S-T1.

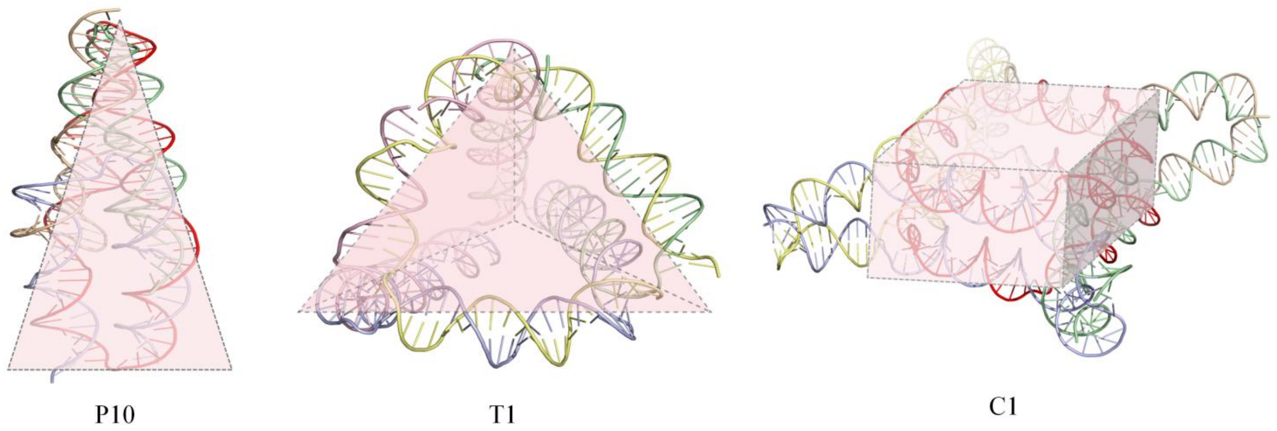

Figure S8 Simulation images of substrates P10, T1 and C1 using AlphaFold 3.

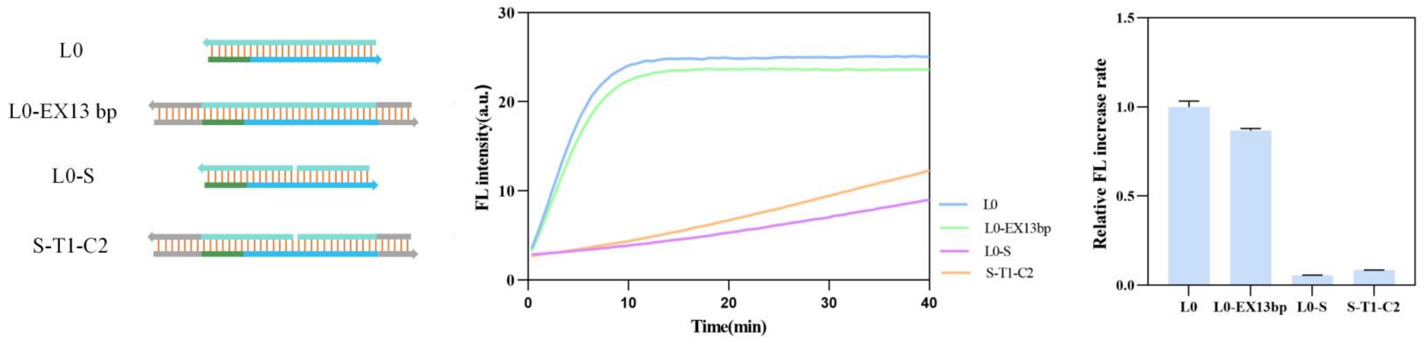

Figure S9 Schematic diagrams of complete and cleaved substrates, along with their fluorescence kinetic curves and relative fluorescence increase rates. All relative fluorescence increase rates are normalized to that of L0. Error bars represent the standard deviation from at least three independent replicates.

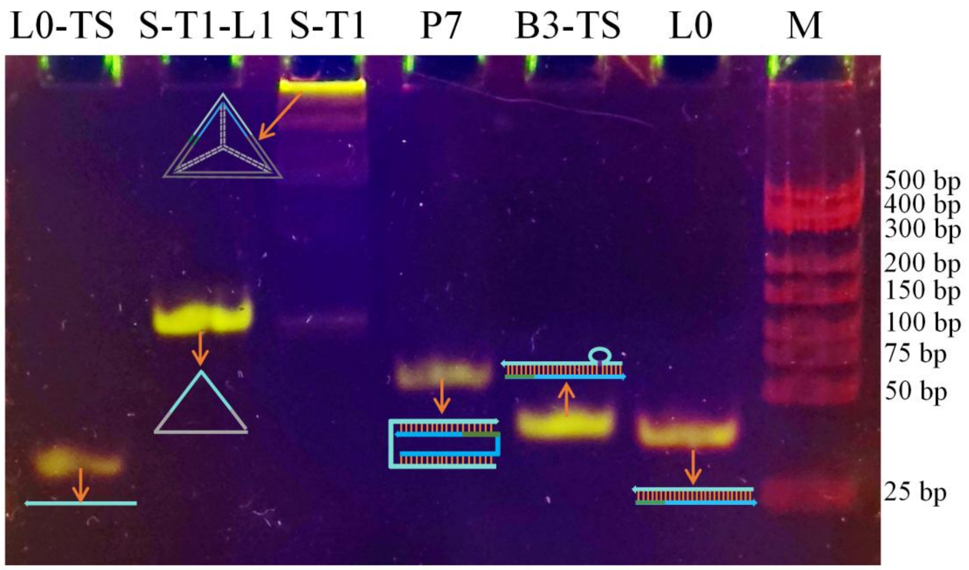

Figure S10 Fluorescence electrophoresis images of various substrates.

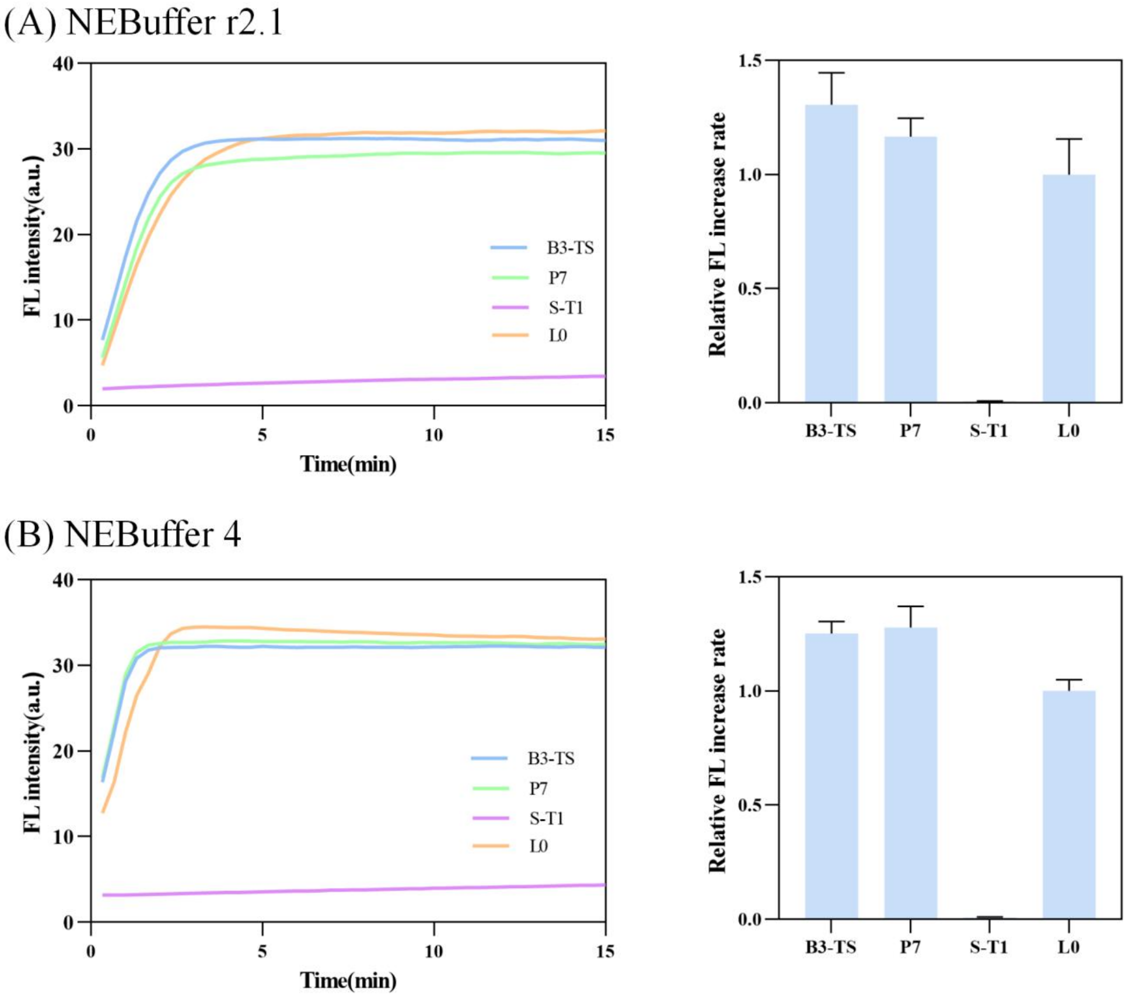

Figure S11 (A) Fluorescence kinetic curves of various substrates in NEBuffer r2.1 and relative fluorescence increase rates. (B) Fluorescence kinetic curves of various substrates in NEBuffer 4 and relative fluorescence increase rates. All relative fluorescence increase rates are normalized to that of L0. Error bars represent the standard deviation from at least three independent replicates.

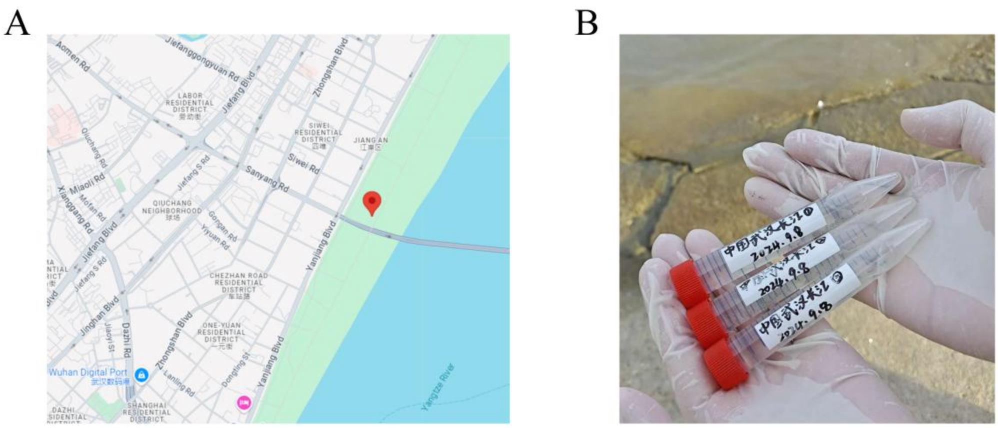

Figure S12 (A) Sampling sites for Yangtze River water samples. (B) There samples from the Yangtze River.

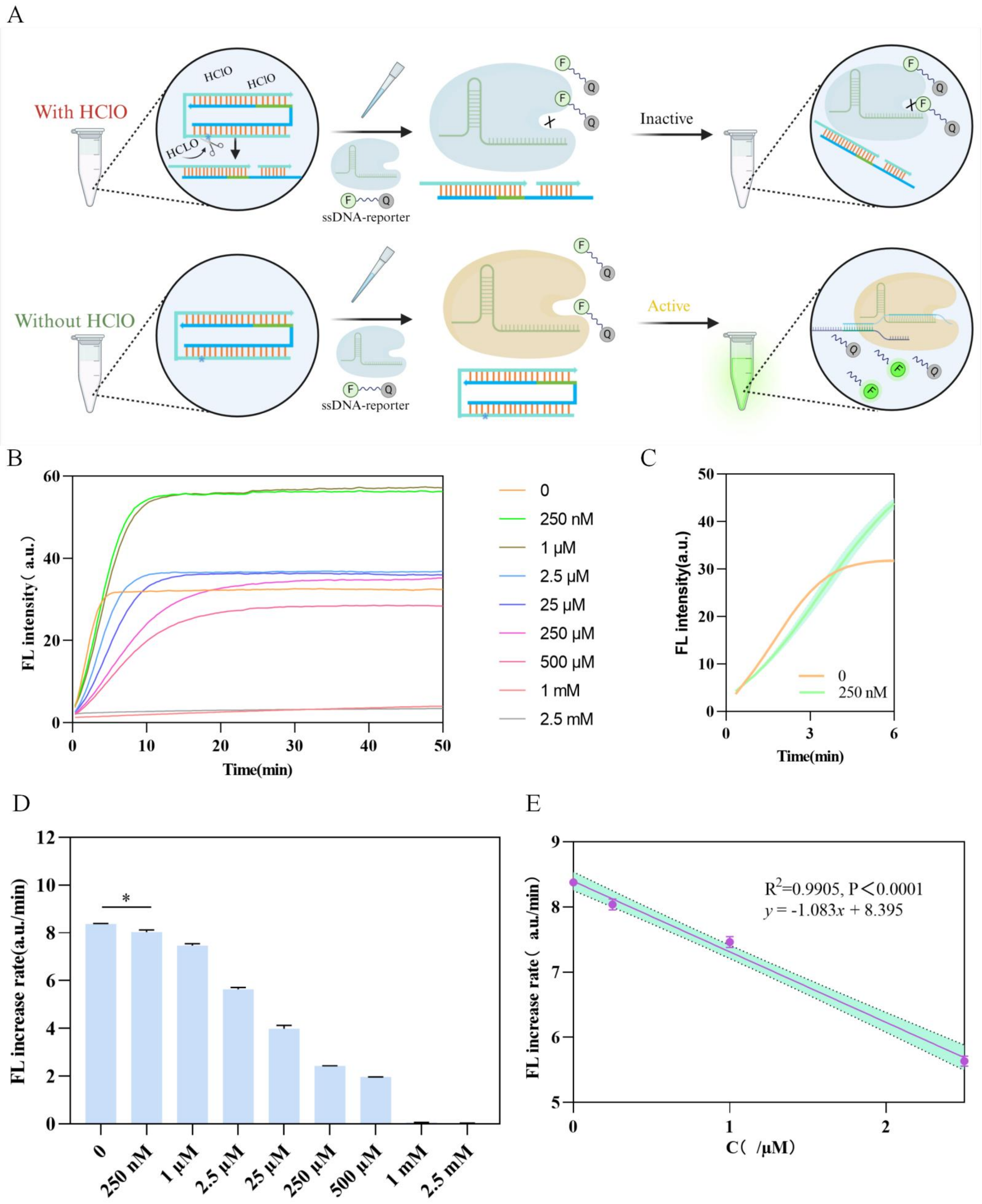

Figure S13 (A)Schematic diagram for detecting HOCl using the substrate P7-20* in "turn off" model. (B) The fluorescence kinetic curves for detecting hypochlorous acid at different concentrations. (C) Enlarged view of the fluorescence kinetic curves for detecting 0 and 250 nM HOCl. (D) The fluorescence increase rates of detecting HOCl at different concentrations. (E) The linear relationship of this detection method. Error bars represent the standard deviation from at least three independent replicates.

Table S2 Comparison between this work and reported methods for hypochlorous acid detection

Analytical method	LOD (/ μM)	Detection Time	Detection strategy
Highly sensitive hypochlorous acid detection system based on Cas12a and topological substrate (This work)	0.088	1.5 h	Cas12a and phosphorothioate DNA
The chemical fluorescent probe method (5)	2.03	0.5 h	Synthesized chemical probe
HPLC and UV-vis spectrometry method (6)	1.00	24.5 h	HPLC and UV
CRISPR/Cas12a-based hypochlorous acid and myeloperoxidase biosensors (7)	0.33	1.7 h	Cas12a and phosphorothioate DNA
UV-vis colorimetric probe (8)	0.81	0.5 h	Chemical probe and UV colorimetry
A functional DNA nanosensor (9)	0.0085	2.2 h	Gold nanoparticle and phosphorothioate DNA

Reference:

1. Swarts, D.C. and Jinek, M. (2019) Mechanistic Insights into the cis- and trans-Acting DNase Activities of Cas12a. Molecular Cell, 73, 589-600.

2. Janice S. Chen, Enbo Ma, Lucas B. Harrington, Maria Da Costa, Xinran Tian, Joel M. Palefsky and Doudna, J.A. (2018) CRISPR-Cas12a target binding unleashes indiscriminate single-stranded DNase activity. SCIENCE, 360, 436-439.

3. Van Dongen, J.E., Berendsen, J.T.W., Eijkel, J.C.T. and Segerink, L.I. (2021) A CRISPR/Cas12a-assisted in vitro diagnostic tool for identification and quantification of single CpG methylation sites. Biosensors and Bioelectronics, 194, 113624.

4. Fang, S., Wang, L., Mei, Y. and Zheng, K. (2022) A ratiometric fluorescent probe for sensing hypochlorite in physiological saline, bovine serum albumin and fetal bovine/calf serum. Spectrochimica Acta Part A: Molecular and Biomolecular Spectroscopy, 269, 120738.

5. Hwang, I., Song, Y.H. and Lee, S. (2025) Enhanced trans-cleavage activity using CRISPR-Cas12a variant designed to reduce steric inhibition by cis-cleavage products. Biosensors and Bioelectronics, 267, 116859.

6. Nejdl, L., Sochor, J., Zitka, O., Cernei, N., Ruttkay-Nedecky, B., Kopel, P., Babula, P., Adam, V., Hubalek, J. and Kizek, R. (2013) Spectrometric and Chromatographic Study of Reactive Oxidants Hypochlorous and Hypobromous Acids and Their Interactions with Taurine. Chromatographia, 76, 363-373.

7. Ma, J., Liu, B., Raza, S., Jiang, H., Tang, A. and Kong, D. (2023) CRISPR/Cas12a-based hypochlorous acid and myeloperoxidase biosensors designed on RESET effect. Sensors and Actuators B: Chemical, 376, 133000.

8. Lou, X., Zhang, Y., Li, Q., Qin, J. and Li, Z. (2011) A highly specific rhodamine-based colorimetric probe for hypochlorites: a new sensing strategy and real application in tap water. Chemical Communications, 47, 3189-3191.

9. Wu, K., Yao, C., Yang, D. and Liu, D. (2022) A functional DNA nanosensor for highly sensitive and selective imaging of ClO^- in atherosclerotic plaques. Biosensors and Bioelectronics, 209, 114273.
